# Pericytes Modulate Third‐Generation Tyrosine Kinase Inhibitor Sensitivity in EGFR‐Mutated Lung Cancer Cells Through IL32‐β5‐Integrin Paracrine Signaling

**DOI:** 10.1002/advs.202405130

**Published:** 2024-10-22

**Authors:** Cheng Huang, Xi Huang, Xiaoyi Qiu, Xiangzhan Kong, Chunmiao Wu, Xue Jiang, Mingkang Yao, Minghui Wang, Liangping Su, Cui Lv, Ping‐Pui Wong

**Affiliations:** ^1^ Guangdong Provincial Key Laboratory of Malignant Tumor Epigenetics and Gene Regulation Guangdong‐Hong Kong Joint Laboratory for RNA Medicine Sun Yat‐sen Memorial Hospital Sun Yat‐sen University Guangzhou 510120 China; ^2^ Medical Research Center Sun Yat‐sen Memorial Hospital Sun Yat‐sen University Guangzhou 510120 China; ^3^ Guangzhou Key Laboratory of Precise Diagnosis and Treatment of Biliary Tract Cancer Department of Biliary‐Pancreatic Surgery Sun Yat‐sen Memorial Hospital Sun Yat‐sen University Guangzhou 510120 China; ^4^ Department of Respiratory Medicine Sun Yat‐sen Memorial Hospital Sun Yat‐sen University Guangzhou 510120 China; ^5^ Department of Thoracic Surgery Sun Yat‐sen Memorial Hospital Sun Yat‐sen University Guangzhou 510120 China; ^6^ Guangdong Provincial Key Laboratory of Urological Diseases Guangzhou Medical University Guangzhou 510120 China; ^7^ Clinical Biobank Center Zhujiang Hospital Southern Medical University Guangzhou 510280 China

**Keywords:** β5‐integrin, non‐small cell lung cancer, pericytes, tyrosine kinase inhibitor sensitivity

## Abstract

EGFR‐mutated lung cancer patients sometimes display restricted responses to third‐generation tyrosine kinase inhibitors (TKIs), potentially attributable to undervalued input from stromal cells, notably pericytes (PCs). The study shows that PCs isolated from EGFR‐mutated patients have a unique secretome profile, notably secreting IL32 and affecting signaling pathways and biological processes linked to TKI sensitivity. Clinical evidence, supported by single‐cell RNA sequencing and multiplex immunostaining of tumor tissues, confirms the presence of IL32‐expressing pericytes closely interacting with β5‐integrin‐expressing cancer cells in EGFR‐mutated patients, impacting therapeutic response and prognosis. Co‐culture and conditioned medium experiments demonstrate that PCs reduce TKI effectiveness in EGFR‐mutated cancer cells, a reversible phenomenon through silencing IL32 expression in PCs or depleting the IL32 receptor β5‐integrin on cancer cells, thereby restoring cancer cell sensitivity. Mechanistically, it is shown that YY1 signaling upregulates IL32 secretion in PCs, subsequently activating the β5‐integrin‐Src‐Akt pathway in EGFR‐mutated cancer cells, contributing to their TKI sensitivity. In animal studies, co‐injection of cancer cells with PCs compromises TKI effectiveness, independently of blood vessel functions, while inhibition of β5‐integrin restores tumor cell sensitivity. Overall, the findings highlight direct crosstalk between cancer cells and pericytes, impacting TKI sensitivity via IL32‐β5‐integrin paracrine signaling, proposing an enhanced therapeutic approach for EGFR‐mutated patients.

## Graphical abstract

This study investigates how pericytes regulate the sensitivity of EGFR‐mutated cancer cells to tyrosine kinase inhibitors (TKIs). It is found that YY1 transcriptionally upregulates IL‐32 secretion in pericytes, which binds to and activates β5‐integrin in cancer cells, triggering the Src‐Akt signaling pathway and reducing TKI sensitivity. Targeting pericytes’ paracrine effect could restore TKI sensitivity in lung cancer.

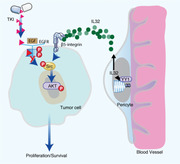

## 1 Introduction

Lung cancer continues to be the primary cause of cancer‐related deaths worldwide, with patients in advanced stages of the disease facing limited treatment choices.^[^ ^1^ ^]^ Non‐small cell lung cancer (NSCLC) accounts for ≈85% of lung cancer cases, presenting a significant challenge due to our incomplete understanding of its underlying pathological mechanisms.^[^ ^2^ ^]^ Recent advancements in next‐generation sequencing have revealed that epidermal growth factor receptor (EGFR) mutations are present in ≈10–35% of NSCLC cases, with some variations based on region and ethnicity.^[^ ^3^ ^]^ EGFR, a transmembrane receptor tyrosine kinase with a central role in governing cancer cell proliferation and survival pathways,^[^ ^4^ ^]^ has become a pivotal therapeutic target for the management of NSCLC tumors, potentially surpassing chemotherapy.^[^ ^5^ ^]^

Considerable progress has been made in developing and clinically evaluating EGFR tyrosine kinase inhibitors (TKIs).^[^ ^3^, ^6^ ^]^ While first‐ and second‐generation TKIs have shown efficacy in treating EGFR‐mutated NSCLC,^[^ ^7^ ^]^ challenges remain due to treatment insensitivity from the emergence of the EGFR T790M mutation and toxicity issues associated with second‐generation TKIs.^[^ ^8^ ^]^ Third‐generation TKIs,^[^ ^8a^ ^]^ like Almonertinib and Osimertinib, have been developed to target EGFR sensitizing and T790M mutations while sparing wild‐type EGFR to reduce toxicity.^[^ ^5^, ^9^ ^]^ Additionally, recent studies have shown that third‐generation TKIs are effective in treating advanced NSCLC patients with EGFR mutations, leading to their approval as a first‐line therapy for this condition.^[^ ^10^ ^]^ However, despite these drugs having been approved for treating NSCLC, some patients still exhibit poor responsiveness,^[^ ^11^ ^]^ with mechanisms not fully understood.

Previous studies on TKI sensitivity have often overlooked the role of the tumor microenvironment, especially stromal cells, in influencing drug response.^[^ ^11b^ ^]^ This oversight may contribute to the limited success of third‐generation TKIs. NSCLC, characterized by aberrant vascularization, fosters a supportive tumor microenvironment promoting cancer progression.^[^ ^12^ ^]^ Traditional approaches targeting tumor vasculature have had limited clinical success,^[^ ^13^ ^]^ potentially worsening tumor hypoxia and reducing chemotherapeutic delivery,^[^ ^14^ ^]^ leading to chemoresistance and metastasis.^[^ ^15^ ^]^ Recent research suggests direct communication between pericytes (PCs) and cancer cells through paracrine signaling, impacting tumor growth and chemotherapy response.^[^ ^12^, ^16^ ^]^ However, the influence of PC‐derived signals on EGFR‐targeted therapy is unknown.

Pericytes, acknowledged for their role in regulating inflammatory responses, particularly during infections, possess the capability to secrete essential proinflammatory factors, which play a vital role in facilitating these crucial processes.^[^ ^16^, ^17^ ^]^ Recent research has indicated that these proinflammatory factors also have a significant impact on propelling cancer growth and progression.^[^ ^18^ ^]^ Among these factors, interleukin 32 (IL32), a well‐known proinflammatory cytokine, has emerged as a substantial contributor.^[^ ^19^ ^]^ IL32 is generated within the tumor microenvironment and has been associated with various aspects of cancer progression.^[^ ^20^ ^]^ Nevertheless, the precise role of IL32 in either promoting or impeding lung cancer cell progression is a topic of ongoing debate. An intriguing aspect is the frequent expression of IL32's known receptor, arginylglycylaspartic acid (RGD) binding integrin (such as β5‐integrin),^[^ ^21^ ^]^ in lung cancer cells.^[^ ^22^ ^]^ Furthermore, there is an active area of research investigating whether IL32 influences cancer cell sensitivity to TKIs and whether PCs are the main source of this cytokine for this specific regulatory purpose.

This study delves into the paracrine influence of pericytes on TKI sensitivity in EGFR‐mutated NSCLC. We found that PCs significantly increase IL32 secretion, which correlates with adverse outcomes in EGFR mutant NSCLC patients. Functional assays highlight PCs' role in regulating cancer cell sensitivity to TKIs, mitigated by depleting IL32 or blocking its receptor β5‐integrin. Mechanistically, YY1 regulates the expression and secretion of IL32 in PCs. IL32 then activates the β5‐integrin‐Src‐Akt pathway, which impairs TKI effectiveness in EGFR‐mutated cancer cells. Co‐injection of cancer cells with PCs reduces tumor responsiveness to TKIs, with IL32/β5‐integrin depletion restoring sensitivity. This underscores the critical role of PC‐secreted IL32 in impacting TKI sensitivity in EGFR mutant NSCLC.

## 2 Results

### 2.1 Pericyte‐IL32 Expression Correlates with Patient Prognosis in EGFR‐Mutated NSCLC Patients

**Figure 1:**
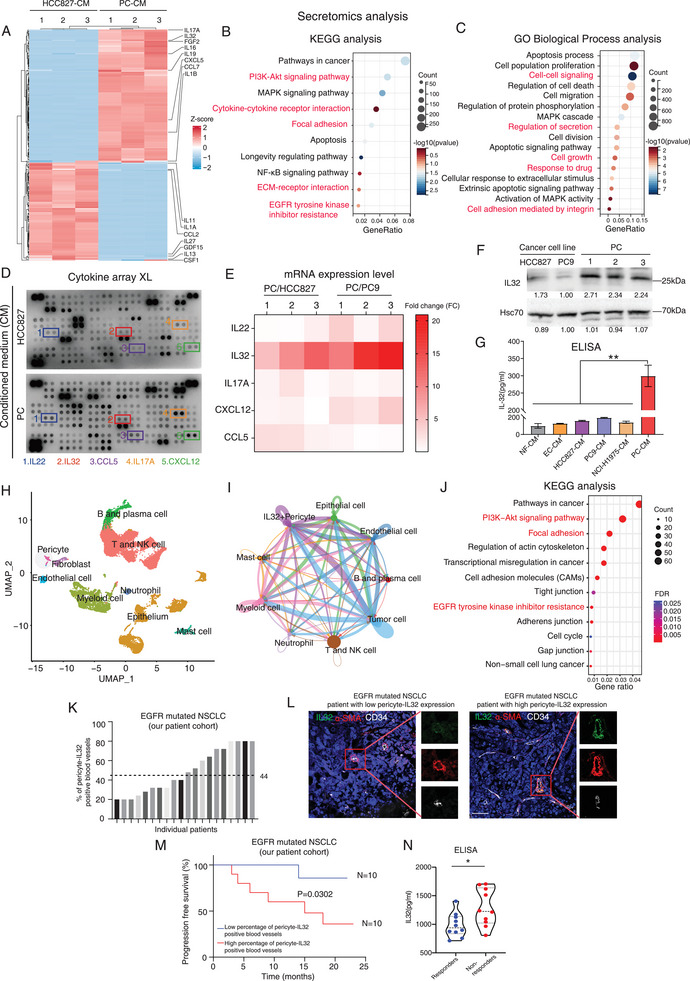
Pericyte‐IL32 expression is associated with cancer progression in NSCLC patients harboring EGFR mutations. A) Visualizing a heatmap comparing secretome analysis between EGFR mutant cancer cell line HCC827 and PCs derived from 3 different NSCLC patients harboring EGFR mutations. Highlighting profound alterations in cytokine secretion in PCs in comparison to cancer cells. B,C) KEGG and GO biological process analyses of the secretomics data in (A). The pathways related to cell‐cell communications and EGFR TKI resistance are highlighted in red. D) Representative images of proteome profiler cytokine XL array from the conditioned medium (CM) of PC and HCC827 cells are given. The expression of IL‐22, IL32, CCL5, IL17A, and CXC12 was up‐regulated in the CM of PCs as compared to that of HCC827 cells. E) Heatmap showing the RT‐PCR analysis of the relative expression of indicated cytokines in PCs as compared to HCC827 or PC9 cells. F) Western blot analysis was performed to evaluate IL32 expression in cancer cells and PCs derived from tumors of three EGFR‐mutated NSCLC patients. The quantification of the Western blot results is provided below the blots. The data are normalized to the control group. G) ELISA measurements were conducted to assess IL32 expression in the CM obtained from normal fibroblast (NF), human umbilical vein endothelial cell (EC), cancer cell lines (HCC827/PC9/NCI‐H1975), and pericyte (PC). H–J) Single‐cell RNA sequencing analysis of the published dataset (GSE171145) derived from NSCLC patients harboring EGFR mutations. UMAP visualization map of all cell type clusters in tumors derived from EGFR mutant NSCLC patients. Each color represents the annotation phenotype of a single cluster (H). The circle plot shows the communication strength between interacting cells. The thicker the line represented, the higher the number of interactions (I). KEGG pathway enrichment analysis in IL32+Pericytes (J). K) The bar chart illustrates the percentage of pericyte‐IL32 positive blood vessels for each patient. L) Representative triple immunostaining images of IL32, α‐SMA, and CD34 on tumor sections from NSCLC patients with EGFR mutations, showcasing low or high pericyte‐IL32 expression, are provided. M) A high percentage of pericyte‐IL32 positive blood vessels correlated with poor progression‐free survival in EGFR‐mutated NSCLC patients (n = 20 patients, our cohort). N) ELISA IL32 assays of serum samples from EGFR‐mutated cancer patients responsive or non‐responsive to third‐generation TKI treatment (n = 20 patients, our cohort). Violin plots represent mean ± S.E.M are given. NS: non‐significant difference. *p < 0.05, ***p < 0.001. (G) One‐way ANOVA. (M) Log‐rank (Mantel‐Cox) test. (N) Student's t‐test. Scale bars in (L) represent 100 µm.

To identify the paracrine factors predominantly secreted by pericytes (PCs) derived from EGFR‐mutated NSCLC patient tumors, we conducted a comparative secretomics analysis of conditioned medium (CM) from PCs derived from three different EGFR‐mutated patients and the EGFR‐mutated NSCLC cancer cell line HCC827. We also compared CM from primary cancer cells with CM from paired PCs isolated from the same three EGFR‐mutated patients (**Figures** **1**A–C; Figures S1A–C, Supporting information). Heatmap analysis of the secretomics data unveiled a distinctive secretome profile in PCs, characterized by altered secretion patterns of cytokines, such as proinflammatory factors IL32 and IL17 family members (Figure 1A; Figure S1A, Supporting information). Both KEGG and GO biological process analyses of the secretomics data indicated an enrichment in signaling pathways and biological processes related to paracrine signaling in PCs, such as PI3K‐Akt signaling pathway, cytokine‐cytokine receptor interaction, EGFR tyrosine kinase inhibitor resistance, ECM‐receptor interaction, cell‐cell signaling, response to drug and regulation of secretion (Figures 1B,C; Figures S1B,C, Supporting Information). To validate our findings, cytokine proteome XL arrays were performed with PCs and HCC827 cells, demonstrating the increased expression of IL22, IL32, CCL5, IL17A, and CXCL12 in PCs (Figure 1D). Further RT‐PCR analysis consistently indicated that IL32 was the only cytokine consistently up‐regulated in PCs when compared to EGFR‐mutated cancer cell lines or their paired primary cancer cells (Figure 1E; Figure S1D, Supporting Information). We also conducted a western blot analysis of IL32 expression in PCs derived from three different EGFR‐mutated NSCLC patients, as compared to cancer cell lines, confirming the up‐regulation of IL32 in all PCs (Figure 1F). To determine the specificity of IL32 up‐regulation in PCs, we performed IL32 ELISA assays on conditioned medium (CM) harvested from normal fibroblasts (NFs), human umbilical vein endothelial cells (ECs), EGFR mutated lung cancer cell lines HCC827, PC9 and NCI‐H1975 (T790M+ cells), and primary PCs, revealing a significant increase in the secretion of IL32 in PC‐CM compared to that from NFs, ECs, or cancer cells (Figure 1G). To further confirm our observation, we also performed RT‐PCR and ELISA analysis with primary cancer cells and paired PCs and their conditioned medium, indicating an up‐regulation of IL32 expression in PCs isolated from 3 different EGFR‐mutated patient‐derived tumors as compared to their paired primary cancer cells (Figures S1D,E, Supporting Information). Further, ELISA assays showed an elevated IL32 level in CM from PCs isolated from EGFR‐mutated patients as compared to CM from PCs isolated from EGFR wild‐type patients (Figure S1F, Supporting Information). These findings provide strong evidence that IL32, an interleukin (IL) associated with tumorigenesis,^[^ ^20a^ ^]^ primarily emanates from PCs within the tumor microenvironment of EGFR‐mutated NSCLC patients.

To assess the clinical significance of our findings, we conducted a single‐cell RNA sequencing analysis of publicly available data (GEO: GSE171145).^[^ ^23^ ^]^ This analysis identified a subset of pericytes within EGFR‐mutated NSCLC patient‐derived tumors that exhibited high IL32 expression (Figure 1H; Figures S1G,H, Supporting Information), predominantly in pericytes compared to other stromal cells like endothelial cells and fibroblasts (Figure S1I, Supporting Information). Further analysis of cell‐cell communications via ligand‐receptor interactions unveiled robust communication between the subset of IL32‐positive pericytes and tumor cells expressing β5‐integrin (gene name: ITGB5) (Figure 1I; Figure S1J, Supporting information). Moreover, this pericyte subset was significantly enriched in pathways related to the PI3K‐Akt signaling pathway, focal adhesion, cell adhesion molecules, and EGFR tyrosine kinase inhibitor resistance (Figure 1J; Figure S1K, Supporting Information). Importantly, β5‐integrin is recognized as the putative binding receptor for IL32.^[^ ^12^, ^20^ ^]^ In contrast, the IL32‐negative pericyte subset exhibited a weaker association with cancer cells compared to the IL32‐positive pericyte subset and did not show enrichment in pathways observed in the IL32‐positive subset, particularly those related to EGFR tyrosine kinase inhibitor resistance. However, the IL32‐negative subset was enriched in pathways associated with metabolic processes (Figures S1L,M, Supporting Information). To further corroborate our observations, we collected a sample cohort of advanced NSCLC patients with EGFR mutations who received third‐generation TKI treatment, with clinicopathological data (Table S1, Supporting Information). We performed triple immunostaining for α‐SMA, CD34, and IL32 on tumor sections to quantify the percentage of pericyte‐IL32 positive blood vessels in each patient, indicating that IL32 was expressed in PCs (Figures 1K,L). Additional immunostaining experiments revealed the expression of β5‐integrin in cancer cells located around blood vessels within EGFR‐mutated tumors (Figure S1N, Supporting Information). Consistently, previous studies showed that β5‐integrin is commonly expressed in various tumors, including NSCLC.^[^ ^24^ ^]^ Our data also indicated that a high percentage of pericyte‐IL32 positive blood vessels correlated with poor progression‐free survival in our patient cohort (Figure 1M). Notably, ELISA assays also showed that serum IL32 levels were higher in non‐responsive EGFR‐mutated patients treated with third‐generation TKIs compared to those who responded (Figure 1N). To corroborate this finding, we utilized the KM plotter database tool to analyze the correlation between the ratio of IL32 expression to pericyte marker expression and overall patient survival. Our results indicated that a high ratio of IL32 expression to pericyte marker expression, such as MCAM, PDGFRB, or ACTA2, was also linked to poor overall survival in NSCLC patients (Figure S1O, Supporting Information). Interestingly, DNA sequencing results revealed that PCs isolated from EGFR‐mutated tumors did not themselves harbor the EGFR mutation (Figure S1P, Supporting Information). This finding aligns with previous reports indicating that stromal cells have a lower mutation burden, and a more stable genome compared to cancer cells.^[^ ^25^ ^]^ In summary, our findings demonstrate that pericyte‐IL32 expression may determine the patient prognosis in EGFR‐mutated NSCLC patients and warrant further functional studies.

### 2.2 Pericytes Reduce TKI Sensitivity in EGFR‐Mutated Cancer Cells through Paracrine Effect

**Figure 2:**
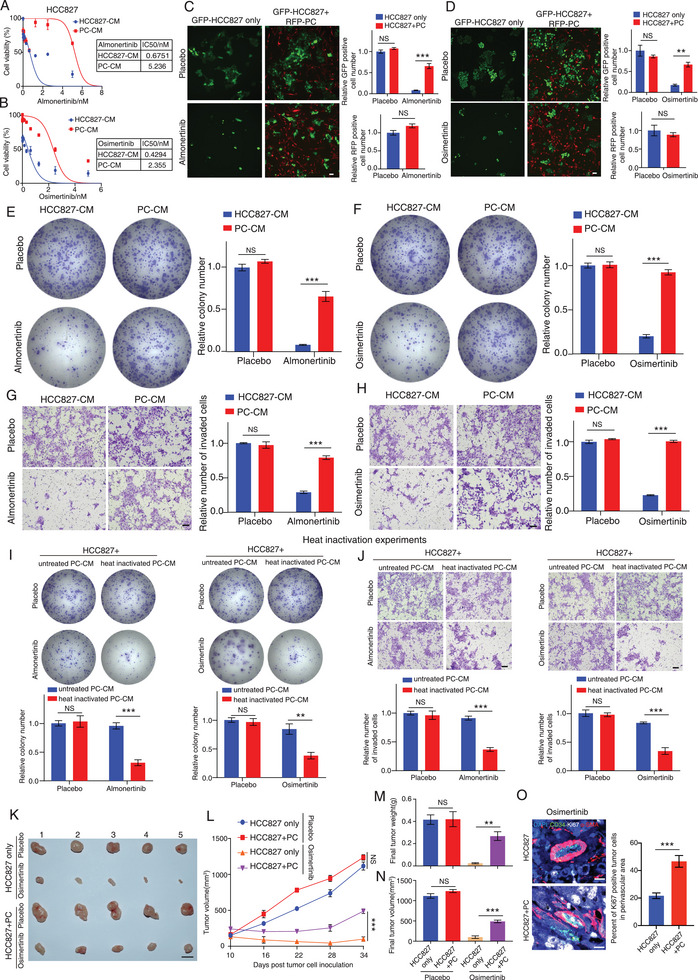
Pericytes modulate the sensitivity of EGFR‐mutated cancer cells to TKIs through a paracrine mechanism. A,B) Osimertinib/Almonertinib IC50 experiments of HCC827 cells treated with conditioned medium (CM) from HCC827 cells or PCs. C,D) Representative images of GFP fluorescently labeled HCC827 cells co‐cultured with/without RFP fluorescently tagged PCs in the presence or absence of Osimertinib/Almonertinib. Bar charts show the relative number of GFP‐positive cancer cells (top) or RFP‐positive PCs (bottom) in each experimental group. E,F) Representative crystal violet‐stained images of colony formation in each group are given. Bar charts show the relative colony number in each group. G,H) Transwell invasion assays of HCC827 cells treated with conditioned medium (CM) from HCC827 cells (HCC827‐CM) or PCs (PC‐CM) in the presence or absence of Osimertinib/Almonertinib. Bar charts show the relative number of invaded cells in each group. I) Representative crystal violet‐stained images of colony formation in each group are given. Bar charts show the relative colony number in each group. J) Representative images of crystal violet‐stained invaded cells of each group are given. Bar charts show the relative number of invaded cells in each group. K–O) Nude mice were subcutaneously injected with either HCC827 cells alone or co‐injected with HCC827 cells and pericytes (PCs) derived from tumors of patients with EGFR‐mutated NSCLC. Tumor‐bearing mice were then treated with a placebo or Osimertinib. Representative gross tumor image from each group is given (K). The line graph represents the tumor growth in each group over time (L). The bar chart shows the final tumor weight (M) or volume (N) in each group (n = 5 mice per group). Representative images of CD34, α‐SMA, and Ki67 triple‐immunostaining on tumor sections derived from each group are given. The violin plot shows the mean percentage of Ki67‐positive tumor cells near blood vessels in each group (O). NS: non‐significant difference. ^**^ *p* < 0.01, ***p < 0.001. C–J,M,N) One‐way ANOVA. C,D (bottom)) Students’ t‐test. L) Two‐way ANOVA. Scale bars in C,D) represent 200 µm. G,H,J) 100 µm. K) 1 cm. O) 10 µm.

Our next objective was to investigate the potential of tumor‐derived pericytes (PCs) in modulating the sensitivity of NSCLC cell lines to TKI treatment. We conducted IC50 experiments with two distinct third‐generation TKI drugs, Almonertinib and Osimertinib, to demonstrate that exposing EGFR mutated cancer cell lines, such as HCC827, PC9 and NCI‐H1975 (T790M+ cell line) to conditioned medium (CM) from PCs resulted in reduced sensitivity to TKI treatment compared to cells exposed to CM from control cancer cells (**Figures** **2**A,B; Figures S2A,B, Supporting Information). Interestingly, our results demonstrated that when EGFR‐mutated cancer cells were exposed to CM from PCs, the effect on the sensitivity of first‐/second‐generation TKI drugs was not as pronounced as the effect on the third‐generation TKI drugs (Figure S2C, Supporting Information). Furthermore, co‐culturing these NSCLC cell lines, labeled with GFP fluorescence, with PCs labeled with RFP fluorescence, also led to decreased sensitivity to TKI drugs compared to the control group, whereas the TKI treatment did not affect PC proliferation (Figures 2C,D; Figure S2D, Supporting Information). Further CCK8 cell proliferation and apoptosis assays indicated that first‐ to third‐generation TKI drugs did not affect PC growth or apoptosis (Figures S2E,F, Supporting Information). Consistently, RT‐PCR analysis showed that the expression of EGFR was lower in PCs as compared to cancer cells (Figure S2G). We then investigated whether exposing these NSCLC cancer cell lines to CM from PCs influenced their colony formation and invasion ability in the presence of TKI drugs. Remarkably, our results revealed that exposure of cancer cells to CM from PCs enhanced their colony formation and invasion abilities in the presence of 3^rd^ generation TKI drugs compared to the group treated with CM from control cells (Figures 2E–H; Figures S2H–J, Supporting Information). Conversely, when cancer cells were exposed to CM from PCs in the absence of TKI drugs, no significant impact on their proliferation, colony formation, or invasion ability was observed, as compared to cells exposed to CM from cancer cells (Figures 2C–H; Figures S2D,H–J, Supporting Information). Additionally, our results showed that exposure to heat‐inactivated CM from PCs could no longer prohibit the inhibitory effect of TKI drugs on the colony formation and invasion ability of HCC827, PC9, and NCI‐H1975 cancer cell lines (Figures 2I,J; Figures S2K–M, Supporting Information). This suggests that PCs may regulate TKI sensitivity in EGFR‐mutated NSCLC cell lines, possibly through paracrine signaling.

To validate our findings in an in vivo setting, we co‐injected HCC827 cells with or without PCs into nude mice subcutaneously. The tumor‐bearing mice received treatment with either placebo or Osimertinib once a week for up to four weeks. Strikingly, our experiments demonstrated that Osimertinib treatment strongly suppressed tumor growth in mice injected with HCC827 cells alone. However, the repressive effect on tumor growth was notably diminished in mice co‐injected with HCC827 cells and PCs (Figures 2K–N). Interestingly, analysis of triple immunostaining experiments showed a higher number of Ki67‐positive tumor cells, particularly around vascular pericytes, in Osimertinib‐treated HCC827 and PC co‐injected tumors compared to Osimertinib‐treated tumors derived solely from the injection of HCC827 cells (Figure 2O). To examine the effect of PCs on cancer cell metastasis in vivo, we co‐injected luciferase‐tagged HCC827 cells with or without PCs into nude mice via the tail vein. Tumor‐bearing mice were treated with either placebo or Osimertinib, followed by bioluminescent imaging for analysis (Figures S2N,O, Supporting Information). The results showed that co‐injection of cancer cells with PCs enhanced experimental lung metastasis as compared to the injection of cancer cells alone (Figures S2N,O, Supporting Information). In summary, our findings suggest that PCs reduce the sensitivity of EGFR‐mutated cancer cells to TKI treatment through paracrine signaling.

### 2.3 Depleting IL32 Expression in PC Rescues its Paracrine Effect on EGFR Mutant Cell Sensitivity to TKI Treatment

**Figure 3:**
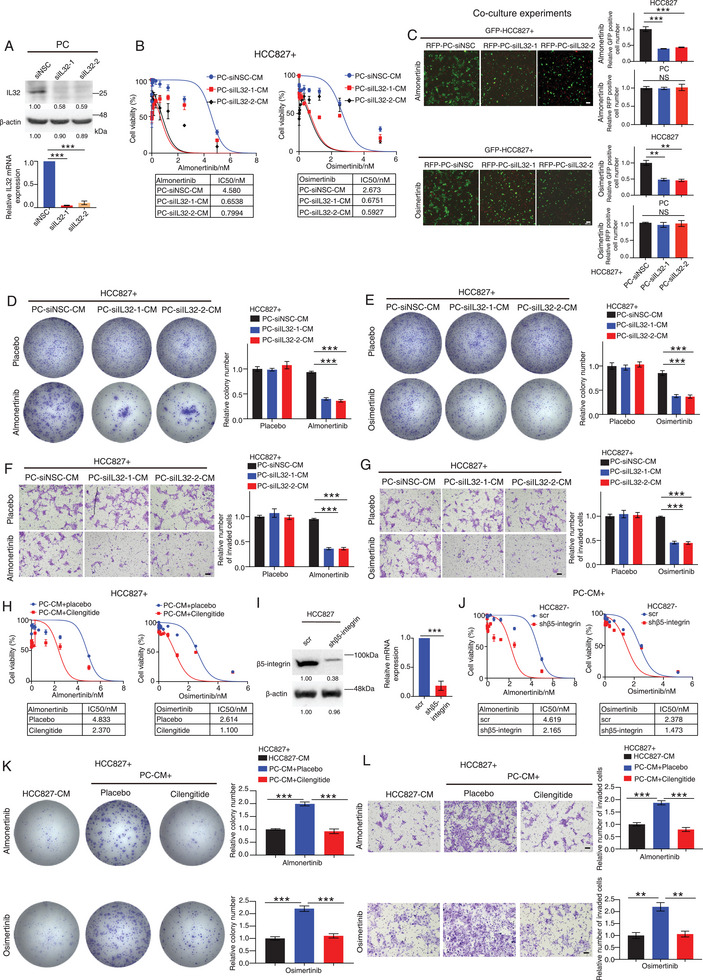
Disrupting IL32‐β5‐integrin paracrine signaling prevents pericyte‐mediated TKI tolerance in EGFR mutated cancer cells. A) Western blot and RT‐PCR analysis of the expression of IL32 in PCs after transfected with IL32 targeting siRNA‐1/‐2 or non‐silencing siRNA (siNSC). The quantification of the Western blot results is provided below the blots. The data are normalized to the control group. B) Almonertinib/Osimertinib IC50 experiments of HCC827 cells treated with conditioned medium (CM) from PCs transfected with either siNSC or IL32 targeting siRNA‐1/‐2. C) Representative fluorescent images of GFP fluorescently labeled HCC827 cells co‐cultured with RFP fluorescently labeled siNSC or siIL32‐1/‐2 transfected PCs in the presence of Almonertinib/Osimertinib. Bar charts show the relative number of GFP or RFP‐positive cells in each group. D,E) Colony formation assays of HCC827 cells after being treated with CM from PCs transfected with either siNSC or IL32 targeting siRNA‐1/‐2 in the presence/absence of Almonertinib/Osimertinib. Bar charts show the relative colony number in each group. F,G) Transwell invasion assays of HCC827 cells after being treated with CM from PCs transfected with either non‐silencing control (siNSC) (PC‐siNSC‐CM) or IL32 targeting siRNA‐1/‐2 (PC‐siIL32‐1/‐2‐CM) in the presence/absence of Almonertinib/Osimertinib. Bar charts show the relative number of invaded cells in each group. H) Almonertinib/Osimertinib IC50 experiments of HCC827 cells after being treated with CM from PCs together with or without Cilengitide. I) Western blot and RT‐PCR analysis of the expression of β5‐integrin in each group. J) Almonertinib/Osimertinib IC50 experiments of HCC827 cells stably transfected with scramble (HCC827‐scr) or β5‐integrin targeting shRNA (HCC827‐shβ5‐integrin) treated with CM from PCs. K) Colony formation assays of HCC827 cells after being treated with CM from HCC827 or PCs in the presence of Almonertinib/Osimertinib together with or without Cilengitide. Representative images of crystal violet‐stained colonies in each group are given. Bar charts show the relative colony number in each group. L) Transwell invasion assays of HCC827 cells after being treated with CM from HCC827 or PCs in the presence of Almonertinib/Osimertinib together with or without Cilengitide. Bar charts represent the relative number of invaded cells in each group. NS: non‐significant difference. ^**^ *p* < 0.01, ***p < 0.001. A,C–G,K,L) One‐way ANOVA. I) Student's t‐test. Scale bars in (C) represent 200 µm. F,G,L) 100 µm.

To determine whether PCs affected EGFR mutant cell sensitivity to TKI through IL32, we knocked down IL32 expression in PCs using two different IL32 targeting siRNAs, and its silencing was confirmed by both western blotting and RT‐PCR (**Figure** **3**A). The transfected cells were then used to perform a range of in vitro tumorigenicity assays. Drug sensitivity experiments showed that exposure of HCC827/PC9 cells with CM from IL32‐depleted PCs reduced their TKI IC50 values when compared to the cells exposed with CM from non‐silencing siRNA (siNSC) transfected PCs (Figure 3B; Figure S3A, Supporting Information). IC50 experiments also demonstrated that the use of an IL32‐neutralizing antibody reversed the repressive effect of CM from PCs on cancer cell sensitivity to third‐generation TKI drugs (Figure S3B, Supporting Information). This observation was further confirmed by the co‐culturing experiment, indicating that co‐culturing EGFR mutant cells with IL32‐depleted PCs reduced the enhanced proliferation observed in the cells after co‐culture with siNSC transfected PCs, whereas there was no difference in the cell number and apoptosis between PCs transfected with either IL32 targeting siRNA‐1/‐2 or siNSC (Figure 3C; Figures S3C,D, Supporting Information). Furthermore, our results showed that exposure of cancer cells with CM from IL32‐depleted PCs reduced their colony formation and invasion ability in the presence of TKI treatment as compared to the cells exposed to CM from scramble transfected cells, whereas it had no effect in the absence of TKI treatment (Figures 3D–G; Figures S3E–H, Supporting Information). Previous studies indicated that IL32 could bind integrin via its RGD motif to regulate breast cancer cell metastasis.^[^ ^21^ ^]^ Notably, β5‐integrin is known to contain the RGD binding domain.^[^ ^12^, ^20^ ^]^ We then investigated whether targeting β5‐integrin in cancer cells could reverse the paracrine effects of PCs on TKI drug sensitivity. Our results indicate that treating HCC827/PC9 cells with CM from PCs, followed by administration of the β5‐integrin inhibitor Cilengitide, or β5‐integrin depletion via shRNA, led to reduced IC50 values for TKI drugs compared to placebo‐treated cells (Figures 3H–J; Figures S3I–K, Supporting Information). Significantly, Cilengitide treatment inhibited the augmented colony formation and invasive potential seen in EGFR‐mutated cancer cells treated with placebo, which were exposed to CM from PCs along with TKI drugs (Figures 3K,L; Figures S3L,M, Supporting Information). In conclusion, our findings suggest that PCs influence the response of EGFR‐mutated cells to TKI treatment, potentially via the IL32‐β5‐integrin signaling pathway. This warrants deeper exploration and investigation.

### 2.4 Pericyte‐Secreted IL32 Activates the β5‐Integrin‐Src‐Akt Cell Survival Pathway, Impacting TKI Sensitivity in Cancer Cells

**Figure 4:**
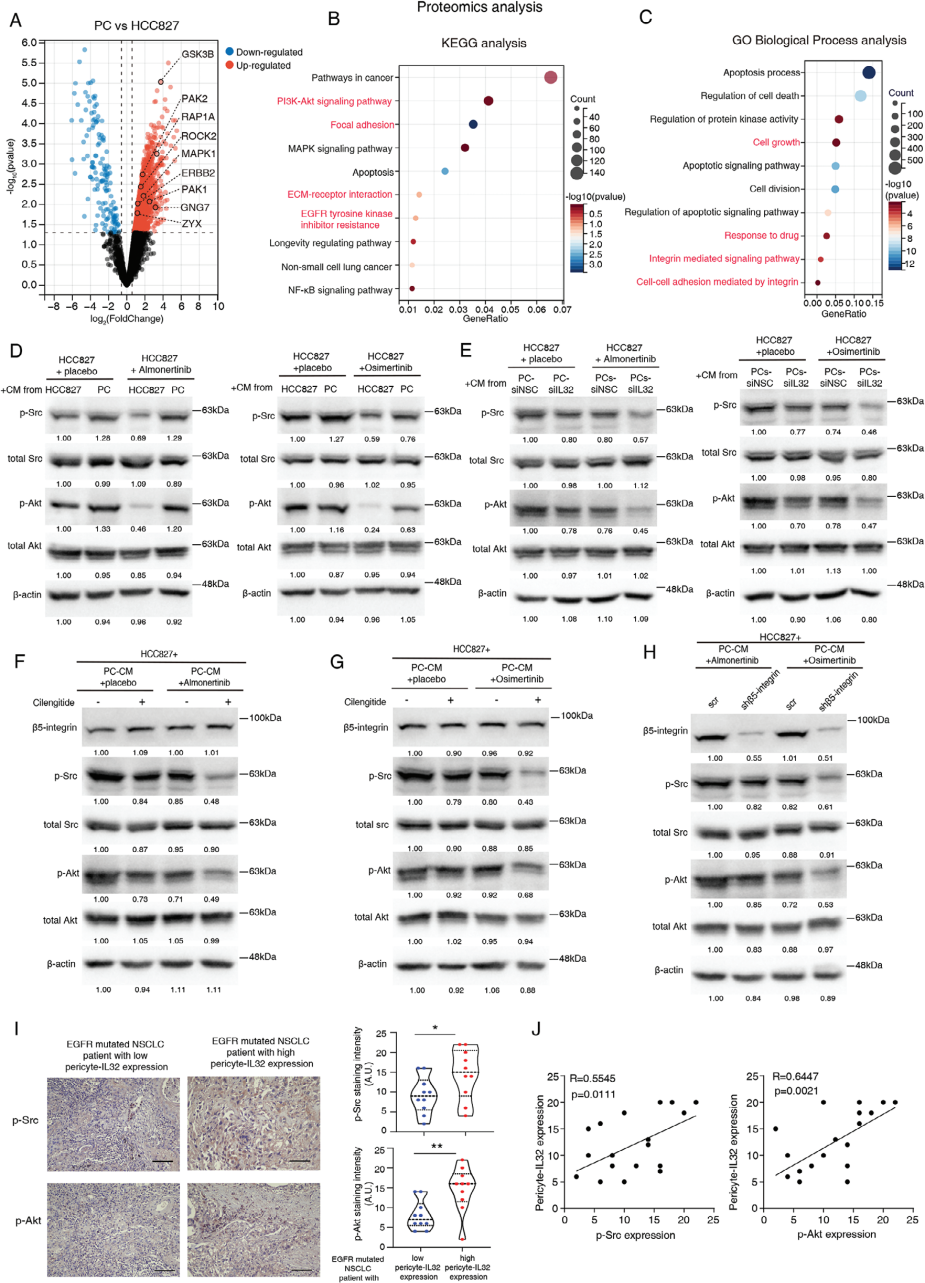
Pericyte‐secreted IL32 regulates β5‐integrin‐Src‐Akt pathway in EGFR mutated cancer cells, influencing TKI sensitivity. A) The volcano plot depicts the proteomics analysis of HCC827 cells following treatment with CM from HCC827 cells or PCs in the presence of Osimertinib. B,C) KEGG and GO pathway analyses of the proteomics data in (A). Pathways related to cell signaling, cell‐cell communications, and drug response are highlighted in red. D) Western blot analysis of the indicated proteins in HCC827 cells after being treated with CM from HCC827 or PCs in the presence or absence of Almonertinib/Osimertinib. The quantification of the Western blot results is provided below the blots. E) Western blot analysis of the indicated proteins in HCC827 cells after exposure with CM from PCs transfected with siNSC or siIL32 in the presence or absence of Almonertinib/Osimertinib. F,G) Western blot analysis was conducted to assess the indicated proteins in HCC827 cells following treatment with or without Almonertinib/Osimertinib, in combination with or without the β5‐integrin inhibitor Cilengitide. H) Western blot analysis was performed on the indicated proteins in HCC827 cells transfected with β5‐integrin‐targeting shRNA (shβ5‐integrin) or scramble shRNA (scr) after being treated with CM from PCs in the presence of Almonertinib/Osimertinib. I) Representative immunohistochemical staining images of p‐Src and p‐Akt in tumor sections from each group are shown. Violin plot shows the staining intensity of p‐Src/p‐Akt in each group (n = 20 patients, our cohort). J) Correlation study between tumor‐p‐Src/p‐Akt and pericyte‐IL32 levels in our EGFR‐mutated NSCLC patient cohort (n = 20 patients, our cohort). I) Student's t‐test. J) Pearson correlation coefficient. Scale bars in (I) represent 100 µm.

To further dissect the underlying molecule mechanism of pericyte‐IL32 mediated TKI sensitivity in cancer cells, we performed comparative proteomics analysis of HCC827 or PC9 cells after being treated with conditioned medium (CM) from either HCC827/PC9 cells or PCs in the presence of TKI treatment. Volcano plot analysis, as well as KEGG and GO biological process analyses of the proteomics data, revealed notable changes and enrichments in protein expression, pathways, and biological processes associated with the PI3K‐Akt signaling pathway, MAPK signaling pathway, EGFR tyrosine kinase inhibitor resistance, response to drug, integrin‐mediated signaling pathways, and cell‐cell adhesion mediated by integrins in cancer cells treated with PC‐conditioned medium alongside the TKI drug, as compared to the control group (**Figures** **4**A–C; Figures S4A–C, Supporting Information). These results suggested that pericyte‐IL32 may affect cancer cell sensitivity to TKI via its receptor integrin‐mediated signaling pathway. Indeed, previous studies have shown that β5‐integrin can regulate cancer cell survival and drug sensitivity through the Src‐Akt pathway.^[^ ^26^ ^]^ We then conducted Western blot analysis on the downstream effectors of β5‐integrin, Src, and Akt, in cancer cells treated with conditioned medium (CM) from PCs or cancer cells, with or without TKI treatment. Our results show that exposing cancer cells to CM from PCs protected them from the inhibitory effects of TKI drugs on Src and Akt phosphorylation and activation, compared to cells exposed to CM from cancer cells (Figure 4D; Figures S4D,E, Supporting Information). Consistently, previous studies indicated that TKI treatment could potentially inhibit EGFR‐mediated Src activation and its downstream Akt‐dependent cell survival/proliferation pathways in EGFR‐mutated cancer cells to reduce their survival, while the expression and activity of Src has been linked with TKI sensitivity.^[^ ^27^ ^]^ In contrast, we demonstrated that exposing cancer cells to CM from PCs did not affect ERK phosphorylation, a key component of the MAPK signaling pathway, regardless of TKI treatment, compared to cells exposed to CM from cancer cells (Figures S4F,G, Supporting Information). To determine whether PCs regulated Src‐Akt signaling in cancer cells via IL32‐β5‐integrin paracrine signaling, we treated cancer cells with CM from PCs transfected IL32 targeting siRNA or siNSC in the presence/absence of TKI drugs. Our results indicated that exposure of cancer cells with CM from siIL32 transfected PCs could no longer repress the inhibitory effect of TKI drugs on Src and Akt phosphorylation as compared to the cells exposed with CM from siNSC transfected PCs (Figure 4E; Figures S4H,I, Supporting Information). Additionally, we showed that treatment with β5‐integrin inhibitor rescued the repressive effect of TKI drugs on Src and Akt phosphorylation in PC‐CM treated cancer cells as compared to placebo‐treated control group (Figures 4F,G; Figures S4J,K, Supporting Information). Furthermore, we demonstrated that suppressing β5‐integrin expression in cancer cells decreased Src and Akt phosphorylation following treatment with CM from PCs in the presence of TKIs (Figure 4H; Figure S4L, Supporting Information), suggesting the specific role of β5‐integrin in mediating cancer cell sensitivity to TKIs. In our clinical observations, immunohistochemistry results revealed a positive correlation between tumor‐p‐Src or p‐Akt and pericyte‐IL32 levels in our EGFR‐mutated patient cohort (Figures 4I,J). To further confirm the effector role of Src in PC‐IL32 mediated TKI sensitivity in cancer cells, we performed IC50 TKI experiments using EGFR‐mutated cancer cells treated with CM from PCs in the presence of TKI with or without Src inhibitor Dasatinib. The results showed that treatment with Dasatinib rescued the sensitivity of cancer cells to TKI treatment in the presence of CM from PCs (Figure S4M, Supporting Information). In summary, our findings indicate that pericyte‐derived IL32 activates the β5‐integrin mediated Src‐Akt signaling pathway, thereby negating the inhibitory effects of TKIs on this pathway in EGFR‐mutated cancer cells.

### 2.5 YY1 Transcriptionally Regulates IL32 Expression and Secretion in PCs

**Figure 5:**
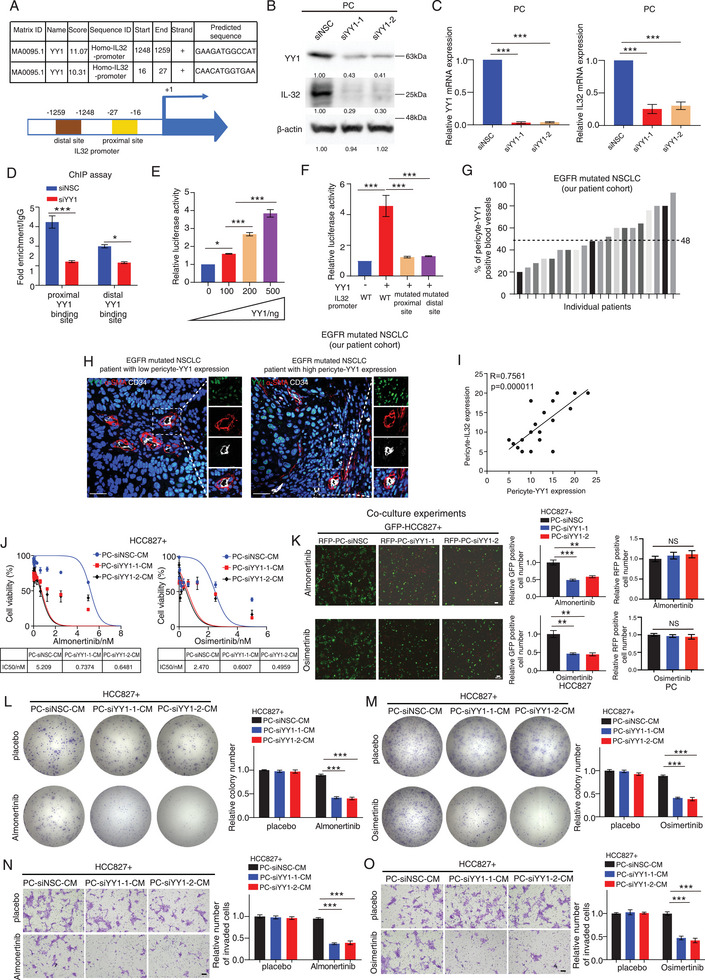
YY1 transcriptionally up‐regulates IL32 expression in pericytes. A) Promoter prediction study of IL32 reveals the presence of transcriptional factor YY1 putative binding sites. Schematic diagram showing the position of the two predicted YY1 putative binding sites. B,C) Western blot or RT‐PCR analysis of YY1 and IL32 expression in PCs transfected with siNSC or siYY1‐1/‐2. The quantification of the Western blot results is provided below the blots. D) Chromatin immunoprecipitation (ChIP) assay showed an increased binding of YY1 to both of its putative binding sites in siNSC transfected PCs as compared to siYY1 transfected PCs. E) The bar chart illustrates the relative luciferase activity in PCs following co‐transfection with a luciferase vector containing the full‐length IL32 promoter sequence and varying levels of YY1 overexpression plasmid. F) The bar chart displays the relative luciferase activity in PCs following co‐transfection with a luciferase vector containing either the full‐length IL32 promoter sequence (WT) or a promoter sequence with mutated proximal or distal YY1 putative binding sites, along with or without a YY1 overexpression plasmid. G) The bar chart shows the percentage of pericyte‐YY1 positive blood vessels in each patient. H) Representative triple immunostaining images of YY1, α‐SMA, and CD34 on tumor sections derived from each group are given. I) Correlation study between pericyte‐YY1 and pericyte‐IL32 expression in our NSCLC patient cohort (n = 20 patients). J) Almonertinib/Osimertinib IC50 experiments of HCC827 cells treated with CM from PCs transfected with either siNSC or YY1 targeting siRNA‐1/‐2. K) Representative fluorescent images of GFP fluorescently labeled HCC827 cells co‐cultured with RFP fluorescently labeled siNSC or siYY1‐1/‐2 transfected PCs in the presence of Almonertinib/Osimertinib. The bar chart shows the relative number of GFP (left) or RFP (right) positive cells in each group. L,M) Colony formation assays of HCC827 cells after being treated with CM derived from siNSC or siYY1‐1/‐2 transfected PCs in the presence/absence of Almonertinib/Osimertinib. The bar chart shows the relative colony number in each group. N,O) Transwell invasion assays of HCC827 cells after being treated with CM derived from siNSC or siYY1‐1/‐2 transfected PCs in the presence/absence of Almonertinib/Osimertinib. The bar chart shows the relative number of invaded cells in each group. NS: non‐significant difference. *p < 0.05, ^**^ *p* < 0.01, ***p < 0.001. C–F,K–O) One‐way ANOVA. I) Pearson correlation coefficient. Scale bars in H,N,O) represent 100 µm. K) 200 µm.

We next explored the molecular mechanism of IL32 up‐regulation in PCs. By performing a transcriptional binding site prediction study with the IL32 promoter, the results indicated that it contains 2 putative YY1 (Yin‐Yang 1), one putative STAT3, and one putative ETS1 transcriptional factor binding site in the IL32 promoter (**Figure** **5**A; Figure S5A, Supporting Information). Interestingly, YY1, STAT3, and ETS1 are transcriptional factors that have been linked with cancer progression and/or inflammatory responses.^[^ ^28^ ^]^ However, their role in regulating pericyte‐IL32 expression and secretion is unclear. We therefore silenced the expression of YY1, STAT3, or ETS1 in PCs by using siRNAs and performed western blot and/or RT‐PCR analysis of the expression of YY1/STAT3/ETS1 and IL32, indicating that the expression of YY1 and IL32 at the protein and mRNA levels was reduced in YY1 depleted PCs as compared to siNSC transfected PCs (Figures 5B,C). In contrast, RT‐PCR analysis showed that silencing STAT3 or ETS1 did not affect IL32 expression in PCs as compared to siNSC transfected PCs (Figures S5B,C, Supporting Information). To further determine the regulatory role of YY1 on IL32 promoter, we performed a chromatin immunoprecipitation experiment using YY1 antibody against siYY1 or siNSC transfected PCs, which were then subjected to RT‐PCR analysis. The results showed increased binding of YY1 to IL32 promoter through its predicted putative binding sites (sites 1 and 2) in siNSC transfected PCs as compared to siYY1 transfected PCs (Figure 5D). Moreover, a luciferase assay revealed that the promoter activity of IL32 in PCs was significantly enhanced in a dose‐dependent manner when co‐transfected with a luciferase reporter vector containing the full‐length IL32 promoter and a YY1 overexpression vector (Figure 5E). Importantly, our results showed that mutating the YY1 putative binding site 1 or 2 significantly reduced the promoter activity of IL32 in PCs (Figure 5F). These results implicated that YY1 transcriptionally regulated IL32 expression in PCs.

To examine the clinical significance of our findings, we also performed triple‐immunostaining of CD34, α‐SMA, and YY1 on tumor sections derived from our EGFR mutated NSCLC patient cohort. Our findings suggested a positive correlation between the expression levels of YY1 and IL32 in pericytes within these patients (Figures 5G–I). To determine the regulatory role of YY1 in PC‐mediated cancer cell sensitivity to TKI drugs, we performed co‐culture experiments with siYY1/siNSC‐transfected PCs and cancer cells. Strikingly, our results showed that exposure of cancer cells with CM from siYY1 transfected PCs reduced their IC50 TKI values as compared to the cells exposed with CM from siNSC transfected PCs (Figure 5J; Figure S5D, Supporting Information) while co‐culturing cancer cells with siYY1 transfected PCs increased their sensitivity to TKI drugs as compared to the group co‐cultured with siNSC transfected PCs (Figure 5K; Figure S5E, Supporting Information). In contrast, knocking down YY1 expression in PCs did not affect their proliferation and apoptosis (Figure 5K; Figures S5E,F, Supporting Information). Furthermore, it also reduced the colony formation and invasion of cancer cells in the presence of TKI drugs as compared to the cells treated with CM harvested from placebo‐treated PCs (Figures 5L–O; Figures S5G–J, Supporting Information). To confirm that IL‐32 is the downstream effector of YY1‐mediated PC effects on EGFR‐mutated cancer cell sensitivity to TKI, we exposed these cells to CM from YY1‐depleted PCs in the presence of TKI drugs, with or without IL32 treatment. The IC50 results indicated that the addition of IL32 increased the IC50 value of TKI drugs in EGFR‐mutated cancer cells treated with CM from YY1‐depleted PCs compared to the placebo‐treated group (Figures S5K,L, Supporting Information). Our findings collectively suggest that YY1 transcriptionally upregulates IL32 secretion from pericytes, influencing their paracrine effect on TKI sensitivity in cancer cells. This provides an initial explanation for the regulatory mechanism of IL32 expression within the tumor microenvironment.

### 2.6 Disrupting Pericyte‐Cancer Cell Crosstalk Rescues the Sensitivity of EGFR Mutated Tumors to TKI, Regardless of Pericyte's Role in Supporting Blood Vessels

**Figure 6:**
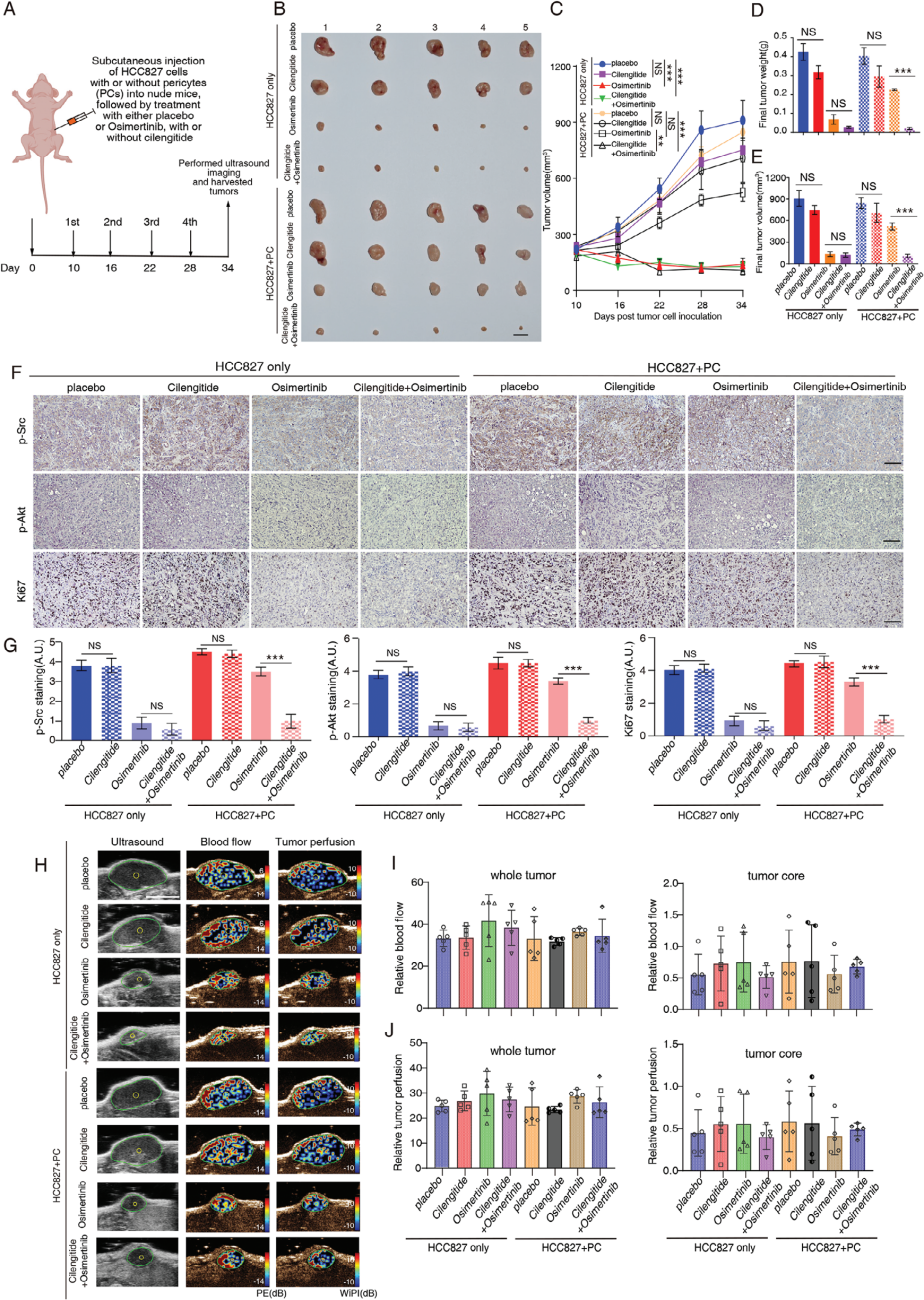
Inhibition of β5‐integrin restores sensitivity to TKI in co‐injected tumors of HCC827 cells and PCs. A) The schematic diagram illustrates the treatment protocol for our animal experiments. Nude mice were subcutaneously injected either with HCC827 cells alone or co‐injected with HCC827 cells and PCs. Tumor‐bearing mice were then treated with either a placebo or Osimertinib, with or without Cilengitide, followed by microbubble‐based ultrasound imaging and tumor harvesting. B) Representative gross tumor image from each group is given. C) The line graph represents the tumor growth in each treatment group. D,E) The bar chart shows the final tumor weight (top) or volume (bottom) in each group. F) Representative immunohistochemistry images of p‐Src, p‐Akt, and Ki67 staining in each experimental group are given. G) Bar charts show the relative staining intensity of p‐Src/p‐Akt/Ki67 in each group. H) Microbubble contrast ultrasound imaging of subcutaneous tumors derived from mice, which were treated with either placebo or Osimertinib. I,J) Bar charts show the relative blood flow or perfusion within the whole tumor or tumor core in each group. NS: non‐significant difference. ^**^ *p* < 0.01, ***p < 0.001. C) Two‐way ANOVA. D,E,G,I,J) One‐way ANOVA. Scale bars in B,H) represent 1 cm. F) 100 µm.

To further examine the regulatory role of pericytes in regulating tumor cell TKI sensitivity in vivo, 4–6 weeks old nude mice were subcutaneously injected with either HCC827 cells alone or co‐injected with HCC827 cells and PCs, which were then treated with placebo or TKI together with or without β5‐integrin inhibitor Cilengitide (**Figure** **6**A). Remarkably, our findings revealed that blocking β5‐integrin with Cilengitide restored the response of tumors derived from co‐injection with HCC827 cells and PCs to TKI treatment, in contrast to the group treated with TKI only (Figures 6B–E). In contrast, the combined treatment with Cilengitide and TKI did not show any additional inhibitory effect on tumor growth in tumors derived from HCC827 cell‐only injections (Figures 6B–E). Moreover, our immunohistochemistry demonstrated a reduction in p‐Src, p‐Akt, and Ki‐67 staining in HCC827 and PC co‐injected tumors treated with Cilengitide and TKI, compared to the group treated with TKI alone (Figures 6F,G). However, there was no difference in the staining intensity of these markers in HCC827‐alone injected tumors treated with Cilengitide and TKI compared to the TKI‐only group (Figures 6F,G). Given the pivotal role of pericytes in regulating blood vessel functions,^[^ ^12b^ ^]^ we conducted in vivo ultrasound microbubble contrast imaging to investigate whether the co‐injection of PCs and tumor cells impacted blood vessel functions and influenced TKI drug sensitivity in vivo. Our results showed no significant difference in blood vessel perfusion and flow between the tumors derived from either co‐injection of PCs and tumor cells or injection of tumor cells alone even in the presence or absence of TKI treatment was observed (Figures 6H–J). To further confirm our observation, we also co‐injected HCC827 cells with PCs transfected with either scramble (scr) or IL32 targeting shRNA (shIL32) subcutaneously. Tumor‐bearing mice were then treated with or without TKI and their tumor growth was monitored over time (Figures S6A–E, Supporting Information). The results indicated that depleting IL32 expression in PCs increased the sensitivity of tumors to TKI treatment as compared to tumors derived from co‐injection of HCC827 cells and scramble transfected PCs (Figures S6A–E, Supporting Information). In contrast, there was no significant difference in tumor growth between HCC827/scramble‐transfected PC and HCC827/shIL32‐transfected PC co‐injected tumors in the absence of TKI treatment (Figures S6A–E, Supporting Information). Overall, these results suggested that PCs conferred TKI sensitivity in EGFR‐mutated tumors through IL32‐β5‐integrin paracrine signaling regardless of their blood vessel supporting role.

In conclusion, we identify PCs regulate EGFR mutant lung cancer cells via a paracrine effect. Clinically, high expression of pericyte‐IL32 is associated with poor prognosis in NSCLC patients harboring EGFR mutations. Mechanistically, YY1 transcriptionally up‐regulates IL32 secretion from PCs, which then activates the β5‐integrin‐Src‐Akt signaling pathway in EGFR‐mutant cancer cells to confer their TKI sensitivity. Our study identifies a promising therapeutic target to enhance TKI responsiveness in NSCLC, highlighting the need for further clinical investigation into its potential efficacy.

## 3 Discussion

NSCLC, a leading cause of global cancer‐related deaths, has seen a shift from traditional chemotherapy to targeted therapies, particularly for EGFR mutations.^[^ ^1^ ^]^ Although first‐generation TKIs show initial efficacy,^[^ ^3b^ ^]^ acquired resistance, often via the T790M mutation, poses challenges.^[^ ^8b^ ^]^ Second‐generation TKIs initially promising against T790M in preclinical studies, encountered dose‐limiting toxicity in clinical trials.^[^ ^7b^ ^]^ Recently, third‐generation EGFR inhibitors like Almonertinib and Osimertinib have emerged, targeting T790M mutations more effectively while preserving wild‐type EGFR function, gaining approval for NSCLC treatment.^[^ ^29^ ^]^ Notably, third‐generation TKIs have also been approved as a first‐line treatment for patients with advanced NSCLC harboring EGFR mutations.^[^ ^10^ ^]^ Despite these advances, some patients remain poorly responsive, with unclear mechanisms,^[^ ^11b^ ^]^ likely influenced by the stromal effect on cancer cell sensitivity to TKI drugs.

Recent research, both from our team and other scientific groups, has shed light on the crucial role of stromal cells, such as pericytes (PCs), in influencing the growth of cancer cells and their response to chemotherapy through paracrine effects, which are independent of their traditional function in supporting blood vessels.^[^ ^12^, ^16^ ^]^ However, the impact of PCs on the sensitivity of cancer cells to targeted therapies remains unclear. To investigate this, we conducted secretomics analysis and cytokine arrays using PC‐derived CM from NSCLC patients with EGFR mutations. We compared these results with those from cancer cells and found a specific increase in the proinflammatory factor IL32 in PCs. Although PCs are commonly recognized as cells that support blood vessels, they are also known to regulate inflammatory responses through their secretome.^[^ ^12^, ^30^ ^]^ In line with this, IL32 has been shown to influence cancer cell survival and apoptosis,^[^ ^21^ ^]^ although the underlying mechanism is unclear. Additionally, its role in modulating EGFR‐mutated cancer cell sensitivity to TKIs has not been explored previously. It was unclear whether PCs are the source of IL32 in the tumor microenvironment and whether this is correlated with cancer progression. Notably, lung cancer cells express the IL32 putative receptor, RGD binding receptor β5‐integrin, which plays a key role in regulating the Src‐Akt pathway,^[^ ^26^, ^31^ ^]^ a known downstream target of TKIs.^[^ ^27^, ^32^ ^]^

Our clinical investigations revealed a strong link between pericyte‐IL32 expression and prognosis in EGFR‐mutated NSCLC patients undergoing third‐generation TKI treatment, suggesting it may indicate their response to treatment. This underscores the clinical relevance of our findings and highlights the potential impact of stromal cells on the efficacy of targeted therapy in NSCLC. Subsequent functional assays showed that PCs could regulate the sensitivity of EGFR‐mutated cancer cells to TKIs through paracrine effects. Depleting pericyte‐derived IL32 or blocking its putative receptor β5‐integrin could reverse their inhibitory effect on TKI sensitivity in cancer cells. Consistent with this finding, RGD binding integrin has previously been linked to the regulation of EGFR‐mutated cancer cell sensitivity to first‐generation TKIs.^[^ ^22^ ^]^ However, the mechanisms underlying its functional role in regulating cancer cell sensitivity to TKIs have remained unclear until now.

To elucidate IL32 upregulation in PCs, we predicted transcriptional binding sites and performed chromatin immunoprecipitation with the IL32 promoter. YY1 was identified as a regulator of IL32 expression in PCs, and YY1 silencing countered the inhibitory effect of PCs on cancer cell TKI sensitivity. Interestingly, YY1, a transcription factor, is highly expressed in various cancers and is implicated in cell survival and apoptosis.^[^ ^33^ ^]^ However, its role in TKI sensitivity has not been investigated until now. Further proteomics analysis revealed enrichment in integrin‐related pathways in cancer cells treated with PC‐conditioned medium and TKIs. PC‐conditioned medium countered TKI effects on downstream EGFR effectors Src and Akt phosphorylation, reversible by Src or β5‐integrin inhibition/depletion, while our animal works also reveal that the co‐injection of cancer cells with PCs diminishes the efficacy of TKIs in limiting tumor burden, irrespective of their blood vessel functions, probably by repressing the inhibitory effect of TKIs on EGFR‐Src‐Akt mediated cell survival/proliferation pathways. Previous studies consistently demonstrate that TKIs target the Src‐Akt signaling pathway, a critical downstream target essential for their impact on cancer cell survival.^[^ ^27^, ^34^ ^]^ Moreover, it is recognized that an unidentified signal triggering Src‐mediated Akt activation influences TKI sensitivity in cancer cells.^[^ ^27c^ ^]^ Our findings demonstrate that PCs can circumvent the inhibitory effect of TKIs on Src‐Akt signaling through IL32‐β5‐integrin paracrine signaling. Interestingly, in comparison to first‐ and second‐generation TKIs, the paracrine effect of PCs on TKI drugs is more distinct in the case of third‐generation TKI drugs. Furthermore, the utilization of the clinically approved Src inhibitor Dasatinib can counteract the suppressive paracrine effect of PCs on the sensitivity of third‐generation TKIs in EGFR‐mutated cancer cells. This highlights the importance of focusing on the role of PCs in modulating the sensitivity of third‐generation TKIs. Additionally, we demonstrate that IL32 depletion or the use of integrin inhibitors can effectively counteract the inhibitory effect of PCs on cancer cell sensitivity to TKI in vivo. This suggests the potential for repurposing integrin/Src inhibitors as a strategy to enhance the efficacy of TKI‐targeted therapy.

## 4 Conclusion

In conclusion, our study reveals the previously unrecognized role of pericytes in modulating the sensitivity of EGFR‐mutated cancer cells to TKI treatment. This discovery urges a reevaluation of our strategy for treating EGFR‐mutated NSCLC patients with elevated pericyte‐IL32 expression. Further clinical investigations are warranted to explore the potential of combining β5‐integrin inhibitor and third‐generation TKIs for these patients.

## 5 Experimental Section

### Ethics Approval and Patient Consent Statement

The collection and usage of patient samples were carried out with the written informed consent of the patients and received approval from the ethical review committee at Sun Yat‐sen Memorial Hospital (SYSKY‐2023‐903‐01). All clinical samples were anonymized in compliance with local ethical guidelines, as mandated by the Declaration of Helsinki. Clinicopathological data are provided in Table S1 (Supporting information).

### Clinical Sample and Data Analysis

All tissue and blood samples from NSCLC patients with EGFR mutations, treated with third‐generation TKIs, were obtained from Sun Yat‐sen Memorial Hospital and confirmed by pathological examination. Pericytes (PCs) were isolated from tumor tissues of patients harboring EGFR mutations, including delE746_A750, L858R, or T790M mutations. Immunostaining followed established protocols.^[^ ^12b^ ^]^ Briefly, paraffin‐embedded tissues were sectioned, de‐waxed, and subjected to antigen retrieval in boiling 10 mm citrate buffer pH 6.0. Sections were washed in PBS and blocked in 1% normal goat serum (NGS) with 0.1% Triton X‐100 for 1 h. Subsequently, sections were triple‐immunostained using anti‐alpha‐smooth muscle actin Cy3‐conjugated (Sigma–Aldrich‐#C6198), anti‐CD34 (Biolegend‐#343610), and either anti‐IL32 (Abcam‐#ab37158), anti‐Integrin β5 (Cell Signaling Technology‐#3629S) or anti‐YY1 antibodies (Cell Signaling Technology‐#46395). After three PBS washes, sections were incubated with secondary antibodies diluted 1:200 in 1% NGS 0.1% TX‐100, washed thrice, and mounted with Prolong Gold Anti‐Fade Mountant with DAPI (ThermoFisher Scientific‐#P36931). For data analysis, the percentage of IL32 or YY1 and alpha‐smooth muscle actin (α‐SMA)‐double‐positive blood vessels was calculated by dividing the number of IL32 or YY1 and α‐SMA‐double‐positive blood vessels by the total number of α‐SMA‐positive blood vessels. Patient data were expressed as those with either lower or higher than the mean percentage of blood vessels positive for pericyte‐derived IL32/YY1 (IL32:44%; YY1:48%).

For KM plotter database analysis, the ratio of IL32 to pericyte marker expression in NSCLC patients was calculated following methods described previously.^[^ ^12b^ ^]^ In brief, this calculation entailed using selected gene probes in the ‘use multiple genes > use ratio of two genes’ option within the KM plotter database tool, which was carried out according to the instructions of the web tool developer.^[^ ^35^ ^]^

### Single‐Cell RNA Sequencing Analysis

Clinical information and data was retrieved from the GEO database (GSE171145),^[^ ^23^ ^]^ specifically selecting samples with EGFR mutations in NSCLC for the analysis. The scRNA‐seq data underwent analysis using the R package “Seurat” (version 4.3.0) in R (version 4.3) as described previously.^[^ ^23^ ^]^ After quality control filtering out low‐quality cells and performing batch effect correction, 22,264 cells and 36,128 genes were left for further analysis. Then, highly variable genes (HVGs) were selected for principal components analysis (PCA), followed by Uniform Manifold Approximation and Projection (UMAP) dimension reduction. Clusters were identified using the FindNeighbors and FindClusters function, the clusters were annotated based on the average expression of curated gene marker sets representing major cell types. The R package “copykat” (version 1.1.0) was utilized to analyze copy number variation (CNV) in the epithelium and identify malignant cells. For cell–cell communication analysis, the R package “CellChat” (version 1.5.0) was employed to explore intercellular interactions, particularly between pericytes and other cell types in the tumor microenvironment.

### Drugs

EGFR‐TKI drugs, including Afatinib, Gefitinib, Osimertinib, and Almonertinib, as well as Cilengitide, were obtained from Selleck Chemicals, while Dasatinib was purchased from Sigma–Aldrich.

### Cell Line and Tissue Culture

HCC827 (RRID:CVCL_2063), PC9 (RRID:CVCL_B260) and NCI‐H1975 (RRID:CVCL_1511) cancer cell lines carry either the EGFR delE746_A750 mutation or the EGFR L858R/T790M double mutation, were procured from the American Type Culture Collection (ATCC) and cultured in RPMI1640 medium supplemented with 10% FBS and 1% penicillin and streptomycin and maintained at 37 °C in a 5% CO_2_ incubator. Human umbilical vein endothelial cells (HUVEC) were isolated and cultured in endothelial cell medium (ScienCell‐#1001) according to the manufacturer's instruction; GFP fluorescently labeled HCC827 and PC9 cells were generated by infecting the cells with GFP overexpressing lentiviruses and subsequently selecting with puromycin (2 µg ml^−1^) until the cells expanded. Pericytes derived from tumor tissues of NSCLC patients with/without EGFR mutations were isolated and cultured following the methodology outlined in a previous publication,^[^ ^12b^ ^]^ and they were maintained in commercially available pericyte medium (ScienCell‐#1201) and cultured at 37 °C in a 5% CO_2_ incubator. DNA sequencing of these PCs to determine the presence of known EGFR mutations was conducted by Guangzhou IGE Biotechnology LTD. The PCs used in the experiments were at a passage number below 8. To obtain mCherry fluorescently labeled PCs, PCs were infected with mCherry overexpressing lentiviral constructs and subsequently selected using puromycin (2 µg ml^−1^). For the heat inactivation experiment, it was done as described previously.^[^ ^36^ ^]^ Briefly, the conditioned media (CM) collected from PCs was subjected to heat treatment at 56 °C for 2 h. Subsequently, the CM was cooled down to 37 °C before being used. The pericyte culture underwent regular mycoplasma testing throughout this study.

### Cell Viability and Apoptosis Assays

The impact of TKI drugs on cancer cell survival or PC proliferation in vitro was assessed using the Cell Counting Kit‐8 assay kit (KeyGen BioTECH‐#KGA317). In brief, HCC827 cells (5000 cells/well), PC9 cells (3000 cells/well), or PCs (3000 cells/well) were seeded in a 96‐well plate containing RPMI 1640 medium supplemented with 10% FBS and 1% penicillin and streptomycin or pericyte medium. The following day, after the cancer cells had adhered to the well, they were exposed to various drug concentrations or control, as specified. For PCs, they were exposed to the IC50 value of each TKI drug, which was determined by using HCC827 cells. After 3 days of treatment or every 12 h within a period of up to 72 h, CCK8 reagent (10 µL/well) was added to the cells and incubated for 2 h before concluding the experiment. Subsequently, the plate was placed in a Tecan Spark® 10M Multimode plate reader to measure the OD value at 450 nm.

For neutralization experiments, a neutralizing antibody against human IL32 (Rockland, USA, 500 ng ml^−1^) or corresponding IgG antibody was pre‐incubated at 37 °C with the supernatant for 1 h before doing IC50 assays. For the Src inhibition experiment, cancer cells were treated with or without 0.1 µm Dasatinib (Sigma–Aldrich‐#SML2589) in the presence of TKI at indicated doses. For the YY1 downstream effector experiment, cancer cells were exposed to CM from siYY1 transfected PCs in the presence of TKI drugs at indicated doses with or without 25 ng ml^−1^ of recombinant human IL‐32 (Biolegend‐#551002). Regarding the apoptosis assays, the proportions of viable cells and apoptotic cells within the entire cell population after indicated treatments were evaluated using a FITC Annexin V apoptosis detection kit with PI (BioLegend‐#6409914).

### Transwell Invasion Experiment

HCC827, PC9, and NCI‐H1975 cells were subjected to serum starvation for 24 h. Subsequently, HCC827 (4.5 × 10^4^ cells/well), PC9 (3.5 × 10^4^ cells/well) or NCI‐H1975 (4.5 × 10^4^ cells/well) were collected and suspended in 500 µL of condition medium. These cells were then seeded into the upper compartments of transwell inserts with an 8 µm pore size (Corning) and coated with a layer of Matrixgel matrix (Corning‐#354230). Following this, 750 µL of RPMI1640 medium supplemented with 10% FBS was added to the lower compartments. After ensuring the absence of bubbles, the transwells were placed in a CO_2_ incubator at 37 °C for 48 h. Subsequently, the chambers and the liquid in the lower compartments were discarded, and 1 mL of 4% PFA per well was added to fix the cells at room temperature for 15 min. The cells were then rinsed with PBS and incubated with 1 mL of 0.1% crystal violet dye at room temperature for 1 h. The chambers were removed using tweezers, and rinsed three times, and any cells that had not migrated beyond the chamber were gently wiped with a cotton swab. The chambers were then inverted to dry, and pictures were taken for subsequent analysis.

### Colony Formation Experiment

HCC827 cells (3000 cells/well) and PC9 cells (2000 cells/well) were initially seeded onto a 6‐well plate and allowed to adhere overnight. Subsequently, they were exposed to 2 mL of conditioned medium derived from HCC827 cells, PC9 cells, or PCs in the presence of vehicle or TKI drugs for 72 h. The cells underwent medium replacement with fresh medium containing either vehicle or TKI drugs twice a week and were continually cultured for up to 10 days. After this period, the cells were washed with PBS twice, fixed with 1 mL of 4% PFA at room temperature for 10–15 min, and treated with 0.1% crystal violet dye for 1 h. Finally, they were washed with 1 mL of ddH_2_O three times, dried, and subjected to imaging under a light microscope to enumerate the number of colonies (with each colony comprising 50 cells) in each experimental condition.

### siRNA/shRNA Transfection

For siRNA transfection experiment, a total of 3.5×10^5^ PCs were seeded in T25 flasks overnight and then transfected with 50 nM YY1/IL32/ETS1/STAT3 targeting siRNA molecules or non‐silencing control siRNA (siNSC) (purchased from IGE bio), using Lipofectamine RNAiMAX (ThermoFisher Scientific‐#13778150) as per the manufacturer's instructions. After 48 h of transfection, both the cells and the culture medium were collected for subsequent analysis. To conduct shRNA experiments targeting β5‐integrin/IL32, specific shRNA sequences designed for this purpose were acquired and integrated them into the psi‐LVRU6P vector obtained from GeneCopoeia. As a comparison, a scrambled shRNA sequence was also included in the psi‐LVRU6P vector. Following transfection of the recombinant constructs along with lentivirus packaging vectors into 293T cells using Lipofectamine 3000, viral supernatant was collected and stored it at −80 °C after filtration. The cancer cells or PCs were then infected with the viruses in the presence of polybrene and subjected to puromycin selection for two weeks. The resulting selected cells were expanded and utilized for subsequent experiments as needed. The sequences of siRNA or shRNA used in this study are given in Table S2 (Supporting information).

### RNA Extraction and RT‐PCR Analysis

Cells were collected and washed with PBS before being transferred to 1.5 mL EP tubes. After centrifugation, the supernatant was discarded, and the cells were resuspended in 1 mL TRIZOL (Biotopped‐#TOP04079) and incubated on ice for 5 min. To each sample, 200 µL of chloroform was added, and the mixture was vigorously shaken for 15 s and allowed to stand at room temperature for 2 min. The samples were then centrifuged at 4 °C, 16000 g, for 15 min. The upper supernatant was carefully transferred to a new EP tube, and an equal volume of cold isopropyl alcohol was added. After gentle mixing, the samples were incubated on ice for 10 min. Following centrifugation at 4 °C, 16000–18000 g, for 10 min, the supernatant was discarded, and the RNA pellet was washed and precipitated with 1 mL of cold 75% ethanol. After another centrifugation at 16000–18000 g for 5 min, the supernatant was removed, and the RNA pellet was air‐dried at room temperature. DEPC water was added to dissolve the samples, and RNA concentration was determined using a Nandrop spectrophotometer. For reverse transcription, 1 µg of RNA template was taken, and 5× All‐in‐one RT SuperMix, Enzyme Mix, and RNase‐free H_2_O (Vazyme‐#R333) were added according to the manufacturer's instructions. The mixture was then centrifuged and subjected to a PCR apparatus for the reaction. The cDNA obtained from reverse transcription was diluted, with generally 20 ng taken as the cDNA template. To this, a 2× SYBR Green mix (Yeasen‐#11201ES08) and the appropriate primers were added, followed by the addition of water. After thorough mixing, the samples were loaded onto the PCR plates and centrifuged. The qPCR analysis was performed using the Roche Light Cycler480 II machine. The sequences of primers used in this study are given in Table S3 (Supporting information).

### Western Blotting

The supernatant from PCs or cancer cells in 10 cm culturing dishes or 6‐well plates was discarded, and the cells were subsequently washed twice with cold PBS. Then, NP40 lysis buffer was added, supplemented with protease inhibitor (Merck Millipore‐#539131, 1:100), and phosphatase inhibitor cocktails (Merck Millipore‐#524625, 1:100). The cells were gently scraped using a cell scraper. Afterward, the lysate was centrifuged at a low temperature, and the supernatant was collected. Protein concentrations were determined according to the instructions of the BCA kit (Invitrogen‐#23225). For further analysis, an equal amount of protein from each sample was heat‐denatured and resolved on 10–12% SDS‐PAGE gels. These gels were then electroblotted onto polyvinylidene difluoride membranes (Merck Millipore‐#3010040001). The membrane was blocked in TBS with 0.1% Tween 20 (TBST) containing 5% milk for 1 h at room temperature. Subsequently, the membrane was probed with the following primary antibodies (diluted 1:1000) overnight at 4 °C: anti‐IL32 (Abcam‐#ab37158), anti‐β5‐integrin (Cell Signaling Technology‐#3629S), anti‐p‐Src (Cell Signaling Technology‐#2101S), anti‐total Src (Cell Signaling Technology‐#2109S), anti‐total Akt (Cell Signaling Technology‐#4691S), anti‐p‐Akt (Cell Signaling Technology‐#4060S), anti‐YY1 antibodies (Cell Signaling Technology‐#46395), anti‐total ERK (Cell Signaling Technology‐#9102S), anti‐p‐ERK (Cell Signaling Technology‐#4370S), anti‐HSC70 (Santa Cruz‐#sc‐7298) and β‐actin (Santa Cruz‐#sc‐47778) (used as a loading control). After washing with TBST, the membrane was incubated with horseradish peroxidase‐conjugated anti‐rabbit secondary antibodies (Cell Signaling Technology‐#7074) for 1 h at room temperature, followed by three washes with TBST. The membranes were visualized using an enhanced chemiluminescence (ECL) system (Merck Millipore‐#WBKLS0500). Western blot quantification was performed using ImageJ software. The data were normalized to the control group.

### Cytokine Array Experiment

The Cytokine array experiment was done using the Proteome Profiler Human XL Cytokine Array Kit according to the manufacturer's instruction (Biotechne‐#ARY022B) and as described previously.^[^ ^37^ ^]^ The conditioned medium from cancer cells or PCs was harvested and used for the experiment.

### ELISA Experiment

The IL32 ELISA was performed according to the manufacturer's instructions (American Research Products Inc.‐#ELK2685). Briefly, 2×10⁴ normal fibroblasts (NF), HUVECs, PCs, HCC827, or PC9 cells were seeded per well in 6‐well plates and incubated overnight. The cells were then cultured in a serum‐free medium, and the supernatant was collected after 48 h for ELISA analysis. Additionally, serum from EGFR‐mutated patients, both responsive and non‐responsive to third‐generation TKI drugs, was tested according to the manufacturer's instructions.

### Animal Model

All animal procedures in this study received approval from the institutional animal care and use committee of Sun Yat‐sen University (SYSU‐IACUC‐2023‐001796). In brief, 4–6 weeks old female nude mice (purchased from Guangdong medical laboratory animal center) were subcutaneously injected with 3×10^6^ HCC827 cells alone or co‐injected with 3×10^6^ HCC827 cells and 1×10^6^ PCs in 100 µL mixture of PBS and Matrigel (BD Biosciences) (1:1 ratio). When the mean tumor size reached ≈200 mm^3^, the tumor‐bearing mice were randomly assigned to different treatment groups, which included: 1. Vehicle alone; 2. Osimertinib alone (administered orally by intragastric injection at a dose of 5 mg kg^−1^ once a week); 3. Cilengitide alone (administered intraperitoneally at a dose of 5mg kg^−1^ once a week); 4. Osimertinib plus Cilengitide. Tumor diameters were measured every 4–5 days using calipers, and tumor volumes were calculated using the formula L×W^2^×0.52, where L represented the tumor length and W represented the tumor width. Subcutaneous tumors from each experimental group were also assessed via ultrasound before euthanizing the mice. For ultrasound imaging and blood vessel function analysis, super‐resolution ultrasound image acquisition was conducted in accordance with the Vevo 2100 ultrasound machine manufacturer's instructions (Visual Sonics), employing the MS250 probe. Ultrasound‐enhanced contrast images were utilized to detect blood flow and tumor vascular perfusion. In brief, microbubble contrast reagents were injected into the tumor‐bearing mice through the tail vein using a tail vein microinjection catheter at 100 µL per mouse. Data analysis was performed using the VevoCQ‐enhanced quantitative software. Throughout the experiments, the animals were kept in controlled conditions, which included maintaining an ambient temperature of 22–24 °C, controlling humidity levels between 40% and 70%, and following a 12‐h light/dark cycle. To investigate the role of IL32 in PC‐mediated TKI sensitivity in cancer cells in vivo, nude mice were co‐injected subcutaneously with 3×10⁶ HCC827 cells and 1×10⁶ PCs transfected with either IL32‐targeting shRNA or scramble shRNA. The tumor‐bearing mice were then randomly assigned to placebo or Osimertinib treatment groups. For experimental metastasis studies, nude mice were injected via the tail vein with 0.7×10⁶ luciferase‐tagged HCC827 cells, with or without 0.3×10⁶ PCs. 10 days post‐injection, the tumor‐bearing mice were then randomly allocated to placebo or Osimertinib treatment groups, and tumor burden was assessed by bioluminescent imaging.

### Immunohistochemistry for Mouse Tumor Tissues

Mouse tumor tissues were fixed in formalin for 24 h and transferred to 70% ethanol. The tissues were then paraffin‐embedded, sectioned, dewaxed, and unmasked in boiling 10 mm citrate buffer pH 6.0. 5 mm sections were washed three times in PBS, blocked in 1% normal goat serum (NGS) 0.1% Triton X‐100 (TX‐100) for 1 h. The tissue sections were immunostained with primary antibodies, including anti‐p‐Src (Cell Signaling Technology‐#2101S), anti‐p‐Akt (Cell Signaling Technology‐#4060S), and anti‐Ki67 (Servicebio Technology‐#GB111499). Subsequently, sections were incubated with enzymatic Avidin–Biotin Complex (ABC) and visualized with 3,3’‐diaminobenzidine (DAB) staining (Vector Laboratories), followed by counterstaining with hematoxylin. Each section was then assigned a score ranging from 0 to 3 based on both the area and intensity of staining. Specifically, the final staining score for each slide was calculated as the product of the area score and the intensity score. For Ki67 staining quantification, it was done as previously described.^[^ ^38^ ^]^ Briefly, Ki67‐positive tumor cells located within a perivascular distance of 50 µm from CD34/α‐SMA double‐positive blood vessels were quantified as a percentage of perivascular DAPI‐positive nuclei.

### Secretomics and Proteomics Analysis

The secretomics and proteomics analyses were conducted as previously described with some modifications.^[^ ^39^ ^]^ For secretomics analysis, HCC827 cells or PCs (2×10^6^ cells/dish) were seeded in a 10 cm culture dish and left undisturbed overnight. Subsequently, the cells were exposed to a serum‐free medium, and after 48 h, their supernatants were collected. These collected supernatants were then subjected to a vacuum apparatus for low‐temperature drying. Subsequently, the dried precipitate was re‐suspended using 50 mm NH_4_HCO_3_ and quantified with a BCA protein quantification kit. From each sample, 50 µg of protein was taken, and four times its volume of acetone was added to precipitate the proteins. The mixture was gently mixed by inversion 6–8 times and then stored at either −30 °C overnight or −80 °C for 2 h. The samples were then removed and centrifuged at 4 °C and 16,000 g for 30 min. The supernatant was discarded, and 500 µL of cold acetone was added to each sample. These were then centrifuged at 16,000g, 4 °C for 5 min. The supernatant was discarded and replaced with 500 µL of cold 70% ethanol (mass spectrometry grade), followed by centrifugation at 16,000 g for 5 min at 4 °C. Finally, the supernatant was discarded and replaced with 500 µL of cold acetone (mass spectrometry grade), followed by centrifugation at 16,000 g, 4 °C for 5 min. After the supernatant was discarded, the sample was placed in a suspension dryer to remove any remaining solvent. The following day, each sample was treated with 50 µL of 8 mm UA buffer, gently agitated with a test tube rack several times, and placed in a mixer at 25 °C, 1300 rpm for 2 h. After centrifugation, each sample was alkylated with IAA in a mixer at 37 °C, 1300 rpm for 2 h. Trypsin (typically 100 µg, corresponding to 2 µL of enzyme) was added to the samples and placed in a mixer at 37 °C, 1300 rpm for 16 h. Subsequently, the samples underwent desalting, vacuum suspension, and peptide concentration determination using a filter solution. Finally, the samples were subjected to secretomics analysis using an Orbitrap Exploris 480 mass spectrometer (ThermoFisher Scientific) with a data‐dependent acquisition mode. The Proteome Discoverer software suite (Version 2.3, ThermoFisher Scientific) was utilized to perform peptide identification and quantification of the samples, as previously described. For proteomics analysis of cancer cells, the main experimental process remained consistent with the secretomics analysis. The distinctions in sample collection and preliminary treatment are summarized as follows: supernatants were discarded, cells were washed twice with PBS, protein lysate was added, and proteins were scraped with a cell scraper. Centrifugation was conducted at 16,000 g at 4 °C for 10 min. The obtained protein supernatant was transferred to a new EP tube, and the protein concentration was determined. Subsequently, 200–300 µg of protein was used for downstream mass spectrometry sample preparation, involving the addition of four times the volume of isopropyl alcohol. The mixture was mixed and then placed in −80 °C for precipitation for 1–2 h. The subsequent procedures were carried out with the supernatant samples.

For secretomics analysis comparing conditioned medium from primary cancer cells with that from paired pericytes (PCs), both were isolated from tumor tissues of patients with confirmed EGFR mutations. Briefly, fresh tumor tissues were washed in saline solution to remove blood. Specimens were micro‐dissected into small pieces (0.5–1 cm^3^). The tissues were enzymatically dissociated at 37 °C for 45 min in a shaking incubator using 5 mL of serum‐free DMEM containing 1 mg mL^−1^ collagenase type 3 (Worthington‐#LS004182) and 1 mg mL^−1^ DNase I (Roche‐#10104159). Digestion was halted by adding an equal volume of DMEM with 10% FBS. The cell suspension was then filtered sequentially through 100 and 40 µm strainers. Cells retained by the 40 µm strainer were resuspended in culturing medium, seeded into 6‐well plates, and cultured undisturbed for 5–7 days. Filtered cells were treated with RBC Lysis Buffer (BioLegend‐#420301) to remove red blood cells, and cancer cells were purified using magnetic‐activated cell sorting (MACS). The tissue pellet was resuspended in 100 µL of MACS separation buffer and incubated with 10 µL of anti‐EpCAM microbeads (Miltenyi Biotec‐#130‐061‐101). EpCAM‐positive cells were magnetically sorted using a MACS column (Miltenyi Biotec‐#130‐042‐401), resuspended in DMEM with 10% FBS, 10 ng mL^−1^ bFGF, 10 ng mL^−1^ EGF and 10 ng mL^−1^ IGF, and cultured in 6‐well plates in a humidified incubator at 37 °C with 5% CO_2_. Additionally, the negative sorted cells were used for PC isolation as previously described.^[^ ^12b^ ^]^ Conditioned medium from primary EGFR‐mutated cancer cells or paired PCs was then harvested for secretomics analysis.

### Statistical Analysis

Statistical analyses in this study were conducted using several methods, including unpaired two‐tailed Student's t‐tests, two‐sided Pearson correlation coefficients, log‐rank (Mantel‐Cox) tests, and One‐/Two‐way ANOVA followed by Tukey's post hoc testing, depending on the data type. The specific statistical tests and exact sample sizes (n) are detailed in the figure legends. All in vitro experiments were independently replicated at least three times. Statistical calculations were performed using GraphPad Prism, with data expressed as means ± SEM. Statistical significance was defined as a p‐value of < 0.05.

### Data Availability

The mRNA sequencing data and clinicopathological information of NSCLC patients were obtained from the website (https://kmplot.com/analysis/index.php?p=service&cancer=lung), following the guidelines provided by the respective platforms and as described previously.^[^ ^40^ ^]^ The proteomics/secretomics data generated in the study had been deposited in the Proteome X consortium via the PRIDE partner repository under accession codes (PXD048904, PXD048950, PXD055731). No new code was created for this study. The data supporting the findings of this study are available in the article and supplementary materials.

## Conflict of Interest

The authors declare no conflict of interest.

## Author Contributions

C.H., X.H., X.Q., and X.K. contributed equally to this work. C.H., X.H., X.Q., and X.K. conducted the majority of experiments and contributed equally to the paper. C.H., X.H., X.Q., C.L., and X.K. performed animal experiments, clinical data, and sample analysis. C.H., X.H., C.W., X.J., and C.L. conducted proteomics and secretomics experiments and analyzed the data. C.H., X.H., C.W., and L.S. performed co‐culture and conditioned medium experiments. M.Y. and M.W. provided clinical samples and technical guidance. M.W., L.S., C.L., and P.‐P.W. co‐conceived the study, supervised the research, and co‐wrote the manuscript. All authors contributed to drafting and approving the final manuscript version.

## Supporting information



Supporting Information

## Data Availability

The data that support the findings of this study are available from the corresponding author upon reasonable request.
