# Supplementary material for: Pericytes Modulate Third‐Generation Tyrosine Kinase Inhibitor Sensitivity in EGFR‐Mutated Lung Cancer Cells Through IL32‐β5‐Integrin Paracrine Signaling
Source: Adv Sci (Weinh). 2024 Oct 22;11(46):2405130. doi: 10.1002/advs.202405130 (PMC11633494; doi:10.1002/advs.202405130)
Supplement: Supplementary file 1 — Supporting Information [file ADVS-11-2405130-s001.docx]

**Supporting Information**

**
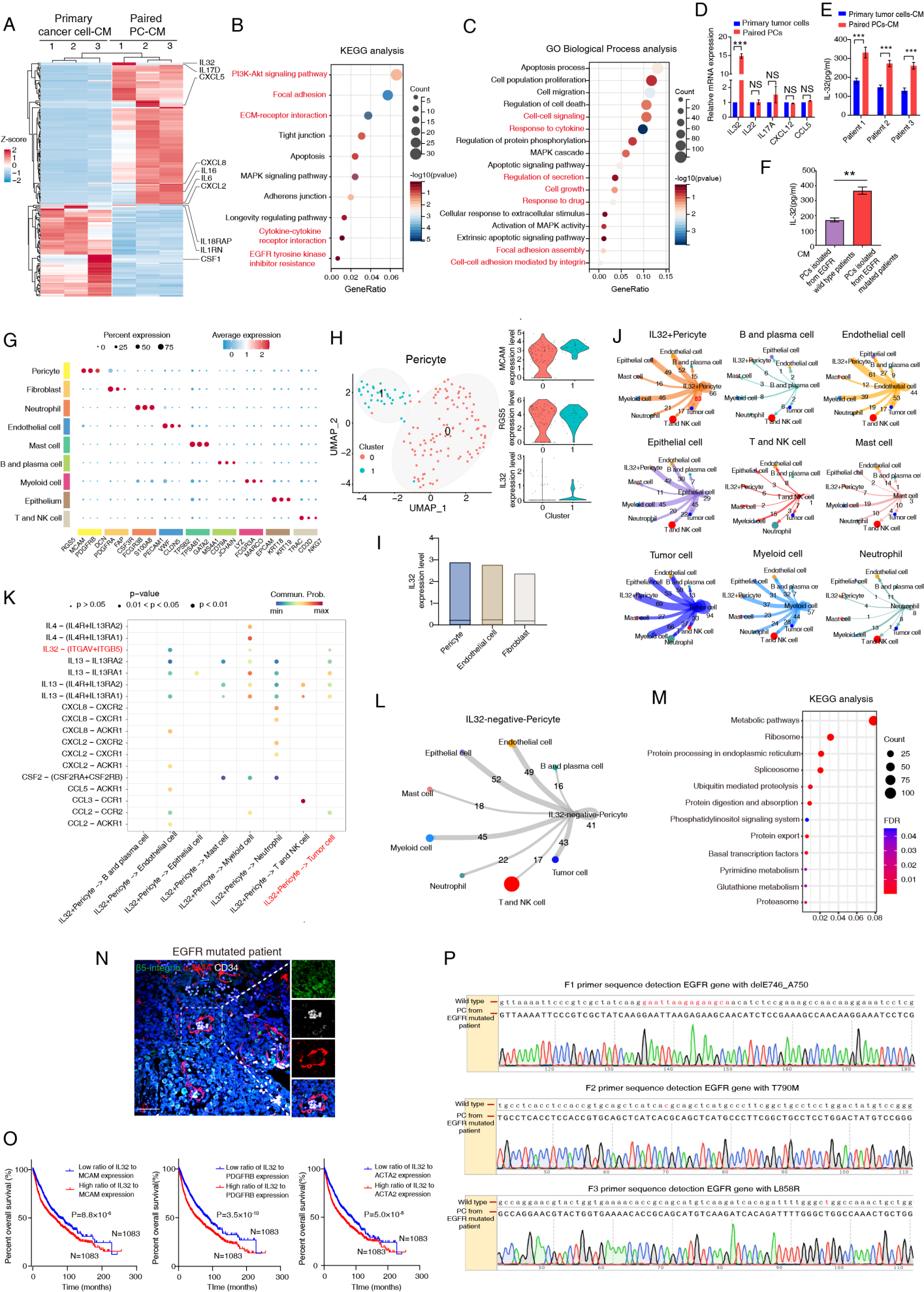
**

**Figure S1. High ratio of pericyte-IL32 positive blood vessels correlates with poor overall survival in NSCLC patients.** (**A**) Secretomics analysis between conditioned medium (CM) obtained from primary cancer cells and paired PCs isolated from 3 different EGFR mutated cancer patients. (**B, C**) KEGG and GO analyses of the secretomics data in (A). (**D**) Bar chart shows the relative mRNA expression of indicated cytokines in PC as compared to paired primary cancer cells (n= 3 independent experiments). (**E**) ELISA measurements were conducted to assess IL32 levels in the conditioned medium (CM) from primary cancer cells and paired PC isolated from 3 different EGFR-mutated cancer patients. (**F**) ELISA analysis of IL-32 levels in the CM from PCs isolated from EGFR wild-type or mutated cancer patients (n= 3 independent samples). (**G**) Dot plots display the average expression of known markers in indicated cell clusters. The size of each dot represents the percentage of cells expressing the genes in each cluster. The intensity of marker expression is also depicted. (**H**) Two pericytes subtypes visualized by UMAP projection (left). Violin plots of marker genes (MCAM and RGS5) for pericytes and IL32 as one of the pericyte subtype marker (right). (**I**) Graph displays IL32 expression levels in pericytes, endothelial cells, and fibroblasts based on single cell sequencing data. (**J**) A detailed perspective is provided on the ligands expressed by each major cell type and the cells that express the signal-receiving receptors. Numbers indicate the quantity of ligand–receptor pairs for each intercellular link. (**K**) Bubble plot shows the ligand–receptor pairs contributing to the signaling from IL32+Pericyte to other clusters. (**L**) Cell-cell communication analysis between IL32-negative pericyte subset and other cell types. (**M**) KEGG pathway enrichment analysis in IL32-negative pericytes. (**N**) Representative triple immunostaining image of β5-integrin, CD34 and α-SMA on tumor section derived from EGFR-mutated cancer patient is given. (**O**) Analysis of the ratio of IL32 to pericyte marker expression in NSCLC patients using the KM plotter database tool (n= 2166 patients). High ratio of IL32 to pericyte marker MCAM (left), PDGFRB (middle), ACTA2 (right) expression associates with poor overall survival in NSCLC patients (n= 2166 patients). (**P**) DNA sequencing was performed to determine whether the isolated PCs from an EGFR-mutated patient carried the delE746_A750, T790M, or L858R mutations. The results presented are representative of three independent samples. NS: non-significant difference. **p < 0.01, ***p < 0.001. (**D, E**) One-way ANOVA. (**F**) Student’s t-test. (**O**) Log-rank (Mantel-Cox) test.


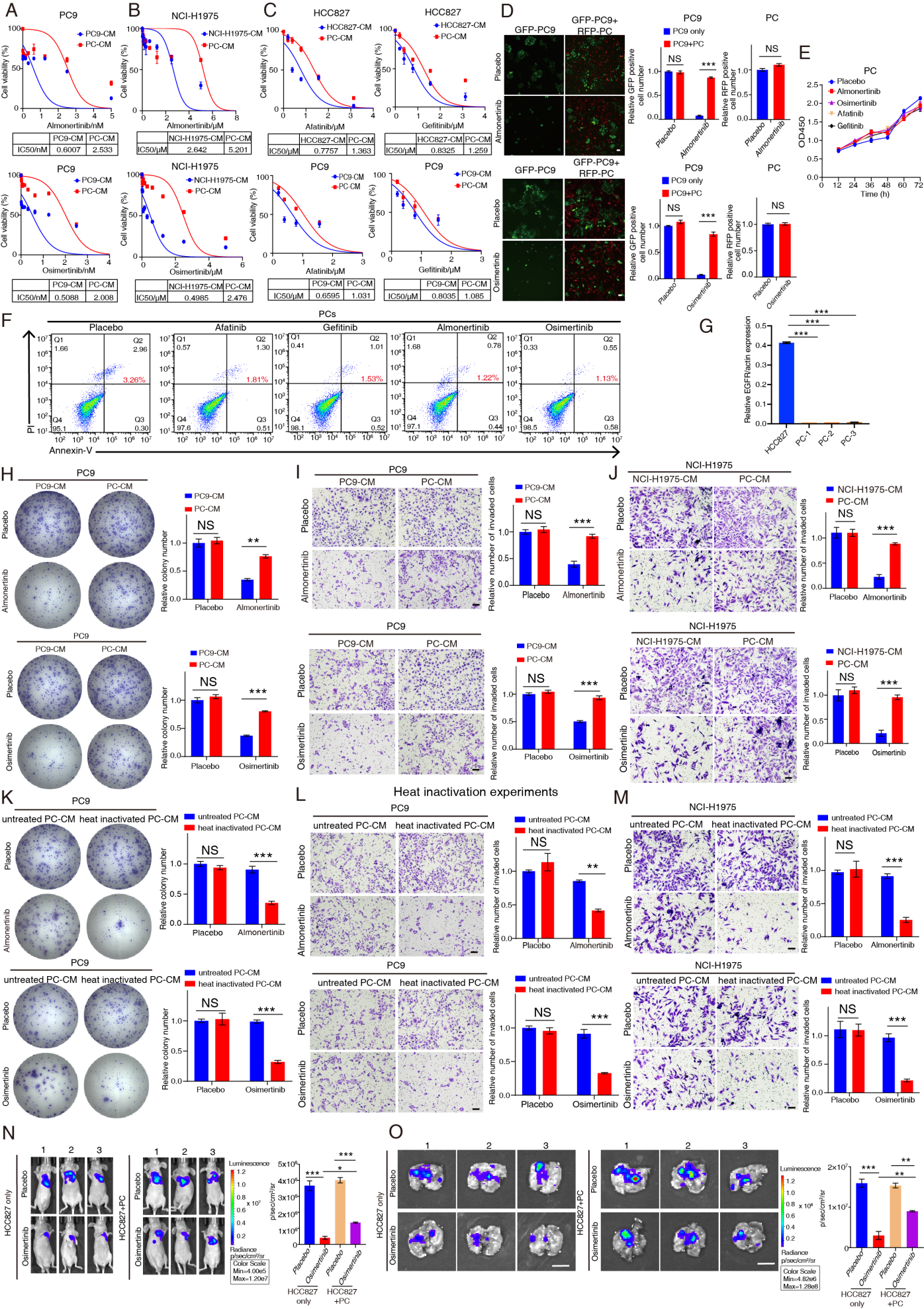


**Figure S2. Pericytes modulate the sensitivity of EGFR-mutant cancer cells to TKIs through paracrine signaling.** (**A, B**) Almonertinib/Osimertinib IC50 experiments were conducted on PC9 or NCI-H1975 cells following treatment with CM from EGFR-mutated lung cancer cell line PC9/NCI-H1975 or PCs. (**C**) Afatinib/Gefitinib IC50 experiments of HCC827/PC9 cells in the presence of CM from HCC827/PC9 cells or PCs. (**D**) Images depict GFP fluorescently labeled PC9 cells co-cultured with/without RFP fluorescently labeled PCs in the presence or absence of Almonertinib/Osimertinib. Bar charts represent the relative number of GFP-positive cancer cells (left) or RFP-positive PCs (right) in each experimental group. (**E**) Line graph illustrates the proliferation of PCs over time following the indicated treatments. (**F**) Annexin V-PI apoptotic assays of PCs following the indicated treatments. The percentages of viable cells, early apoptotic cells, and late apoptotic cells were determined based on the lower left quadrant (Q4), lower right quadrant (Q3), and upper quadrant (Q2), respectively. The red numbers indicate the percentage of apoptotic cells in each group. (**G**) Bar chart shows the relative EGFR/actin expression in each group. (**H**) Images show representative colonies stained with crystal violet in each group. Bar charts display the relative colony number in each group. (**I, J**) Transwell invasion assays were performed on PC9/NCI-H1975 cells treated with CM from PC9/NCI-H1975 cells or PCs in the presence or absence of Almonertinib/Osimertinib. Bar charts illustrate the relative number of invaded cells in each group. (**K-M**) Colony formation and/or transwell invasion assays were conducted on PC9/NCI-H1975 cells following exposure with heat inactivated or untreated CM from PCs in the presence/absence of Almonertinib/Osimertinib. (**N, O**) Nude mice were injected via tail vein with luciferase-tagged HCC827 cells, either alone or with PCs, followed by treatment with placebo or Osimertinib. The mice were then subjected to bioluminescent imaging. Representative in vivo bioluminescent images of tumor-bearing mice from each group at the final time point are shown. The bar chart displays the bioluminescence intensity for each group (N). Representative ex vivo bioluminescent images of harvested lungs from each group are shown, along with a bar chart indicating the bioluminescence intensity for each group (O). NS: non-significant difference. *p<0.05, **p < 0.01, ***p < 0.001. (**D, G-O**) One-way ANOVA. (**D** (right)) Student’s t-test. Scale bars in (**D**) represents 200 μm. (**I, J, L, M**) 100 μm. (**O**) 1 cm.


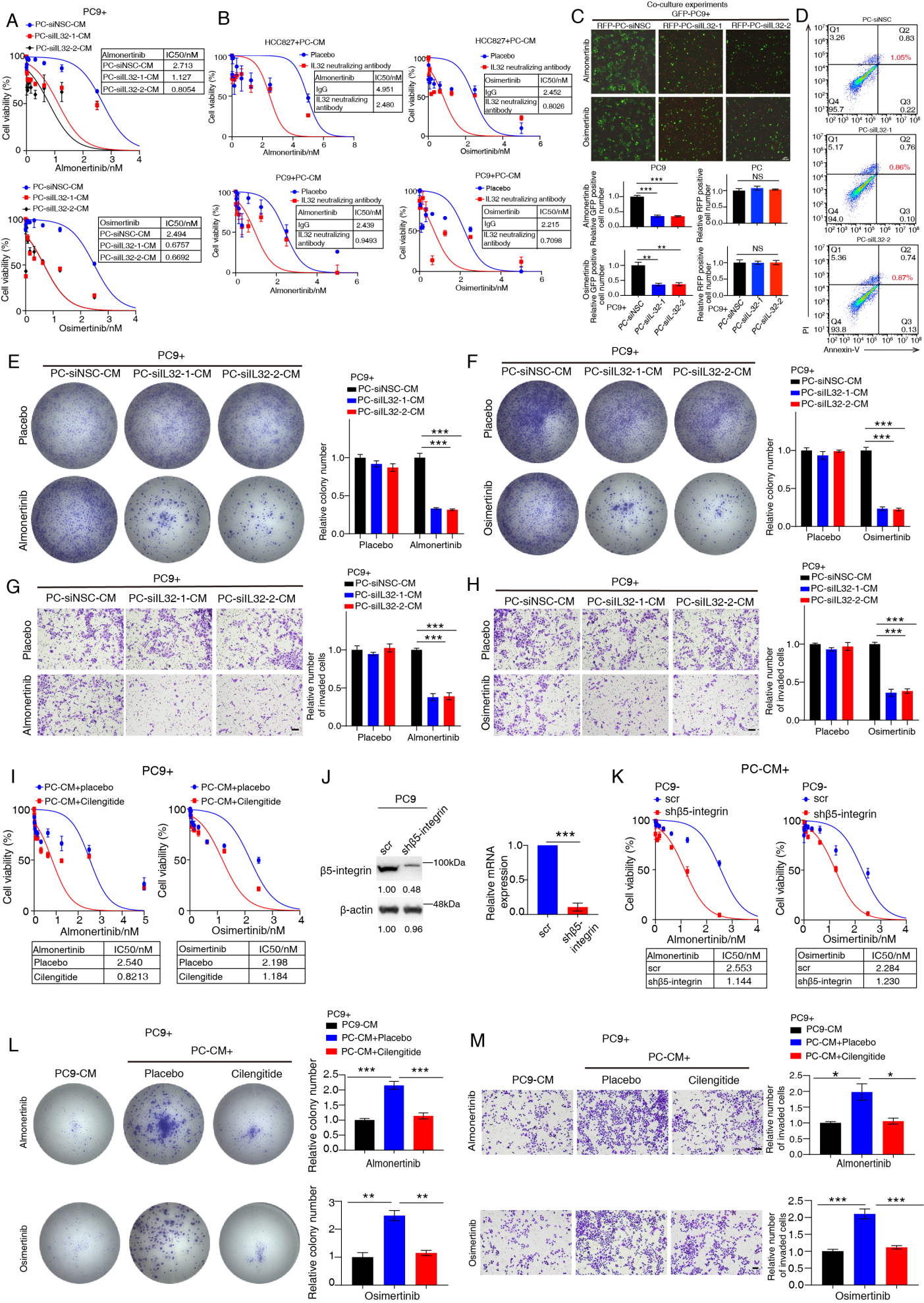


**Figure S3. Blocking IL32-β5-integrin paracrine signaling reverses the inhibitory effect of pericytes on EGFR mutant lung cancer cell sensitivity to TKIs.** (**A**) Almonertinib/Osimertinib IC50 experiments conducted on PC9 cells treated with CM from PCs transfected with either siNSC or IL32 targeting siRNA-1/-2. (**B**) Almonertinib/Osimertinib IC50 experiments conducted on PC9 and HCC827 cells treated with CM from PCs in the presence of IgG or IL32 neutralizing antibody. (**C**) Representative fluorescent images displaying GFP labeled PC9 cells co-cultured with RFP labeled siNSC or siIL32-1/-2 transfected PCs in the presence of Almonertinib/Osimertinib. (**D**) Annexin V-PI apoptotic assays of PCs after transfected with siNSC or siIL32-1/-2. (**E, F**) Colony formation assays of PC9 cells following treatment with CM from PCs transfected with either siNSC or IL32 targeting siRNA-1/-2 in the presence/absence of Almonertinib/Osimertinib. (**G, H**) Transwell invasion assays of PC9 cells after exposure to CM from PCs transfected with either siNSC or IL32 targeting siRNA-1/-2 in the presence/absence of Almonertinib/Osimertinib. (**I**) Almonertinib/Osimertinib IC50 experiments on PC9 cells following treatment with CM from PCs with or without Cilengitide. (**J**) Western blot and RT-PCR analysis of β5-integrin in PC9 cells stably transfected with scramble or β5-integrin targeting shRNA. (**K**) Almonertinib/Osimertinib IC50 experiments conducted on PC9 cells stably transfected with scramble or β5-integrin targeting shRNA treated with CM from PCs. (**L**) Colony formation assays for PC9 cells treated with CM from PC9 or PCs in the presence of Almonertinib/Osimertinib, either with or without Cilengitide. Representative images of crystal-violet-stained colonies for each group are provided. (**M**) Transwell invasion assays of PC9 cells exposed to CM from PC9 or PCs in the presence of Almonertinib/Osimertinib, with or without Cilengitide. NS: non-significant difference. *p < 0.05, **p < 0.01, ***p < 0.001. (**C, E-H, L, M**) One-way ANOVA. (**J**) Student’s t-test. Scale bars in (**C**) represents 200 μm. (**G, H, M**) represent 100 μm.


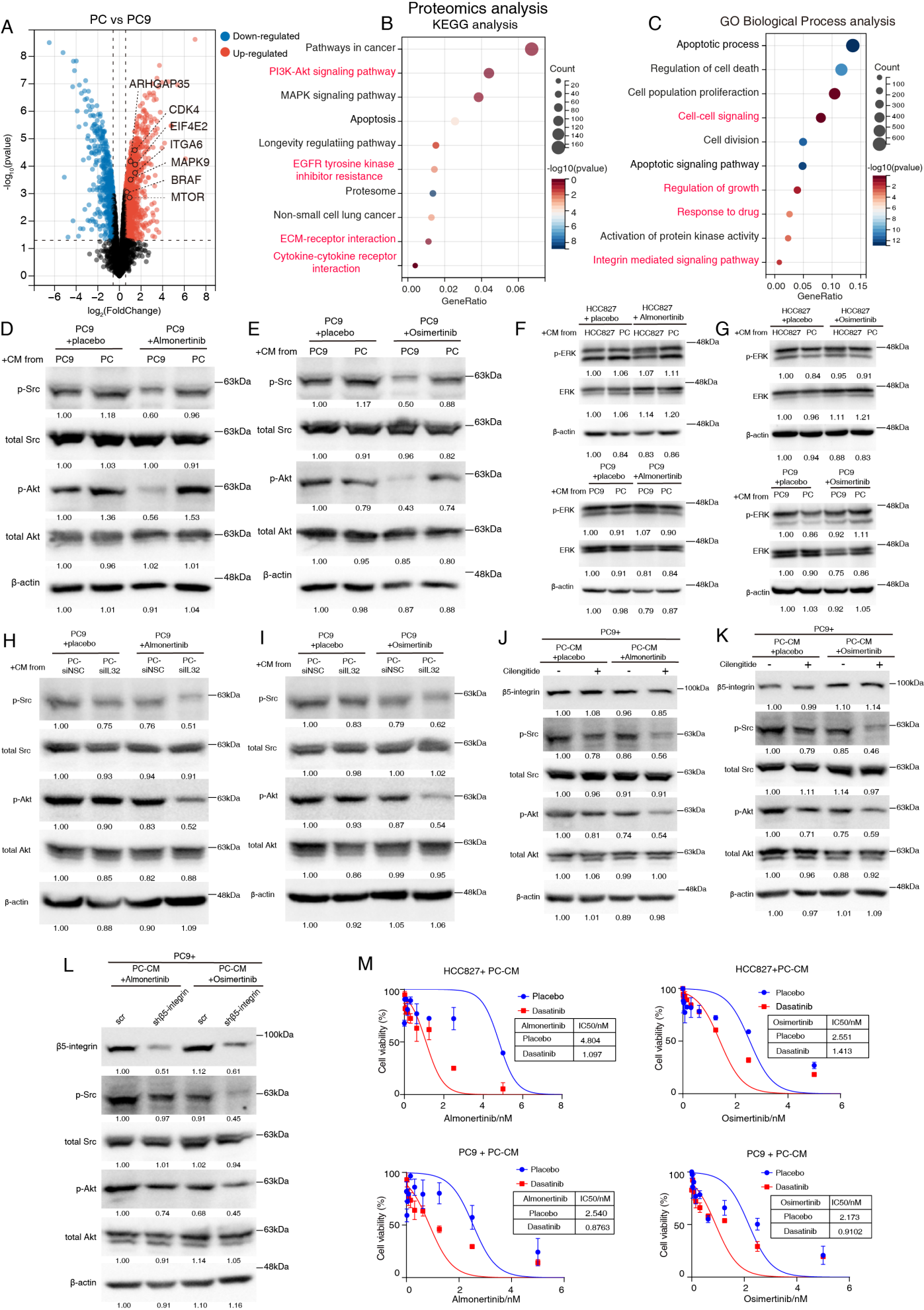


**Figure S4. Pericyte-IL32 diminishes the TKI-induced repression of the β5-integrin-Src-Akt signaling pathway in EGFR mutant lung cancer cells.** (**A**) Volcano plot illustrates the differentially expressed proteins in PC9 cells treated with conditioned medium (CM) from PC9 cells or PCs. (**B, C**) KEGG and GO biological process analyses were conducted on the proteomics data from (A). (**D, E**) Western blot analysis of the indicated proteins in PC9 cells after treated with CM from PC9 cells or PCs in the presence or absence of Almonertinib/Osimertinib. The quantification of the Western blot results is provided below the blots. (**F, G**) Western blot analysis of the indicated proteins in HCC827 and PC9 cells after treated with CM from HCC827 or PC in the presence or absence of Almonertinib/Osimertinib. (**H, I**) Western blot analysis of PC9 cells after exposed with CM from siNSC/siIL32 transfected PCs in the presence/absence of Almonertinib/Osimertinib. (**J, K**) Western blot analysis of the indicated proteins in PC9 cells after treated with CM from PCs in the presence or absence of Almonertinib/Osimertinib, together with or without Cilengitide. (**L**) Western blot analysis of the indicated proteins in PC9 cells stably transfected with scramble/β5-integrin targeting shRNA after treated with CM from PCs in the presence of Almonertinib/Osimertinib. (**M**) Almonertinib/Osimertinib IC50 experiments were conducted on HCC827 and PC9 cells treated with CM from PCs in the presence/absence of Dasatinib.

**
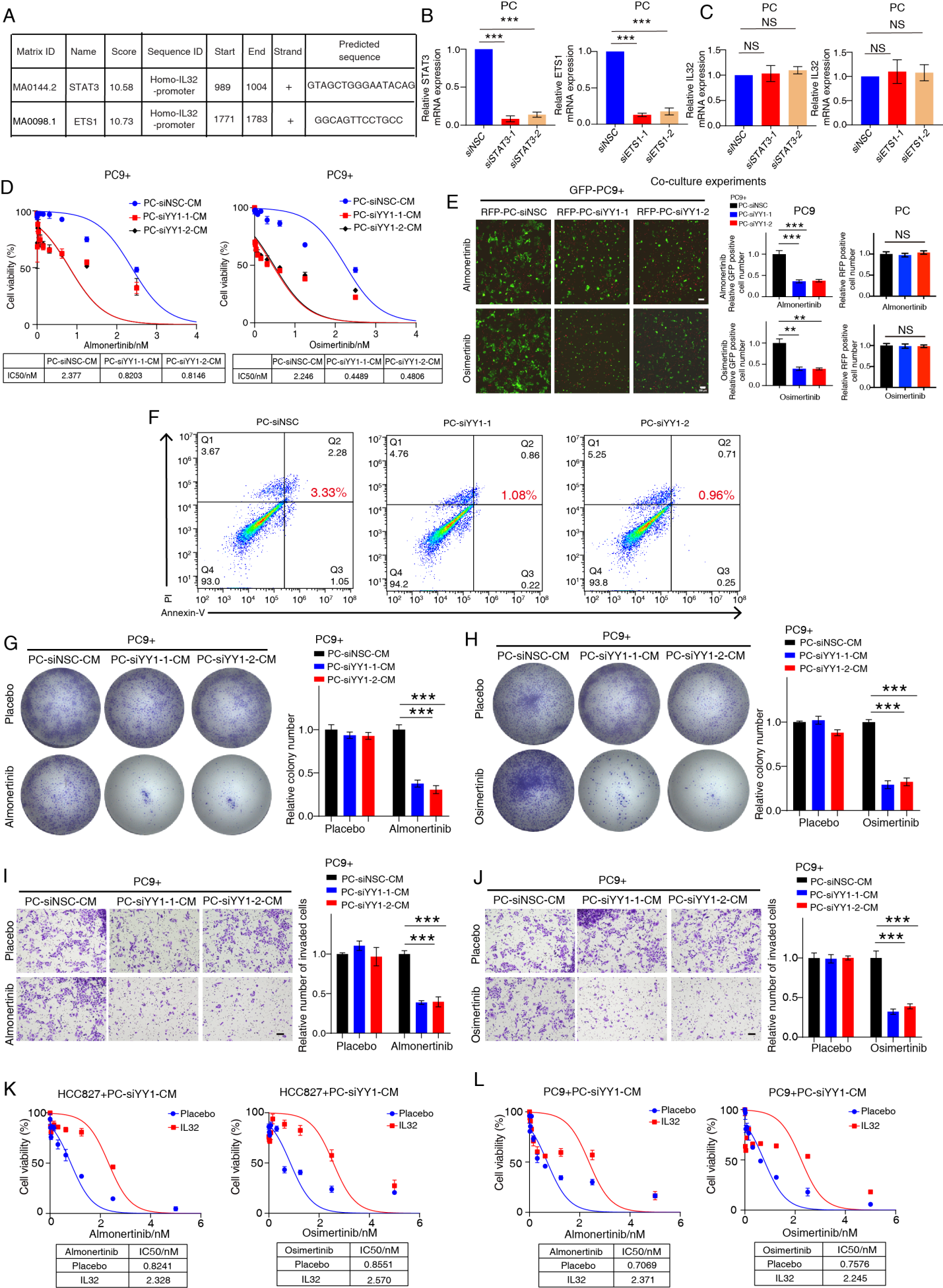
**

**Figure S5. YY1 depletion prohibits PC mediated EGFR-TKI sensitivity in EGFR mutant lung cancer cells.** (**A**) Table presents the promoter prediction study of IL32, revealing the presence of putative binding sites for the transcription factors STAT3 and ETS1. (**B**) RT-PCR analysis of STAT3 or ETS1 expression in PCs transfected with siNSC, siSTAT3, or siETS1-1/-2. (**C**) RT-PCR analysis of IL32 expression in PCs transfected with siNSC, siSTAT3, or siETS1-1/-2. (**D**) IC50 experiments of Almonertinib/Osimertinib in PC9 cells treated with CM from PCs transfected with siNSC or YY1-targeting siRNA-1/-2. (**E**) Representative fluorescent images of GFP labeled PC9 cells co-cultured with RFP-labeled siNSC or siYY1-1/-2 transfected PCs in the presence of Almonertinib/Osimertinib. Bar charts show the relative number of GFP (left) or RFP (right) positive cells in each group. (**F**) Annexin V-PI apoptotic assays of PCs after transfected with siNSC or siYY1-1/-2. (**G, H**) Colony formation assays of PC9 cells treated with CM derived from siNSC or siYY1-1/-2 transfected PCs in the presence/absence of Almonertinib/Osimertinib. Bar chart shows the relative colony number in each group. (**I, J**) Transwell invasion assays of PC9 cells treated with CM derived from siNSC (PC-siNSC-CM) or siYY1-1/-2 transfected PCs (PC-siYY1-1/-2-CM) in the presence/absence of Almonertinib/Osimertinib. Bar chart shows the relative number of invaded cells in each group. (**K, L**) Almonertinib/Osimertinib IC50 experiments of HCC827/PC9 cells after treated with CM from PC transfected with siYY1 in the presence of placebo or IL32. NS: non-significant difference. **p < 0.01. ***p < 0.001. (**B, C,** **E,** **G-J**) One-way ANOVA. Scale bars in (**E**) represents 200 μm. (**I, J**) represent 100 μm.


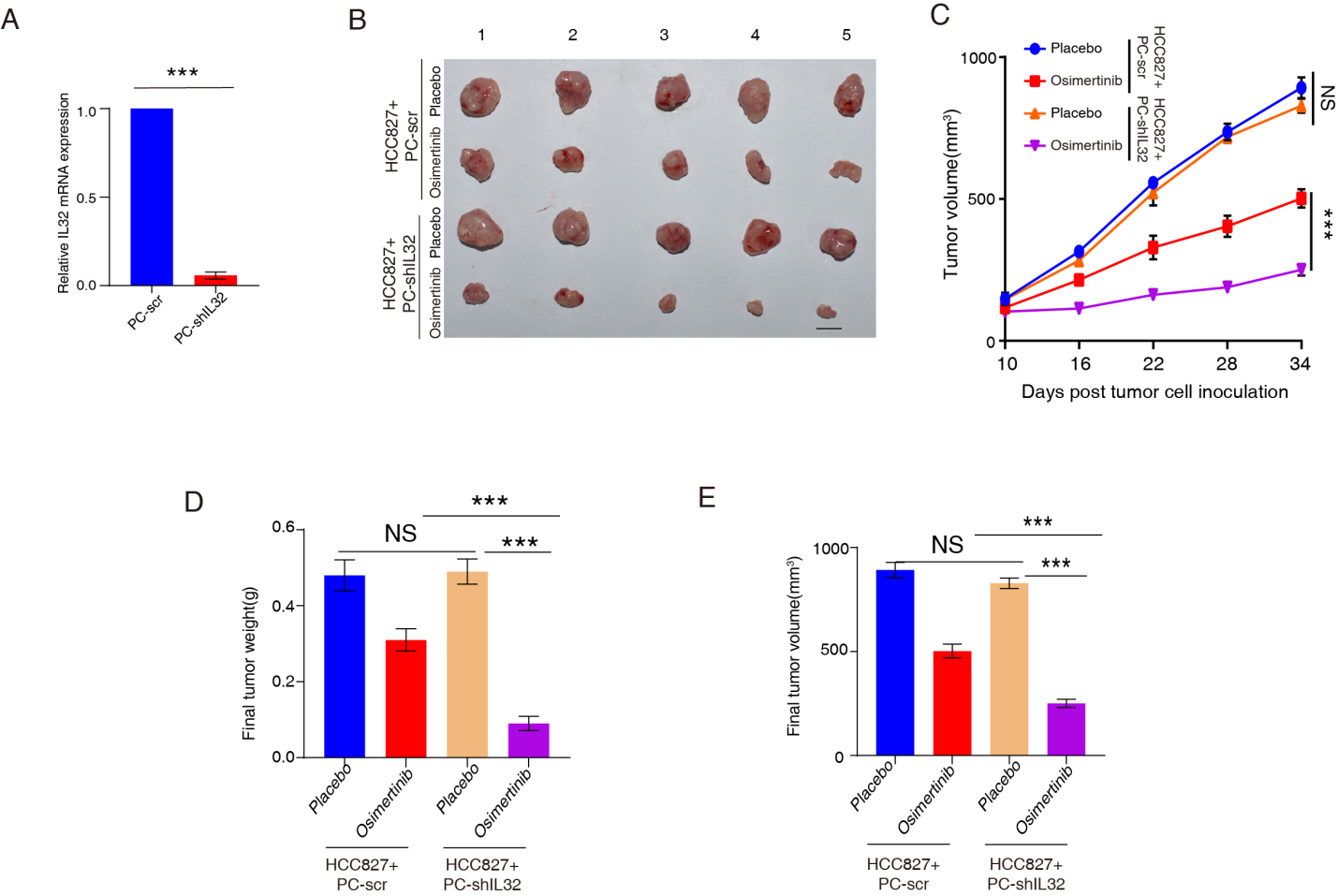


**Figure S6. IL32 depletion in pericytes diminishes their paracrine effect on TKI sensitivity in EGFR-mutated cancer cells in vivo.** (**A**) Bar chart shows the relative expression level of IL32 in PCs stably transfected with IL32 targeting shRNA as compared to scramble shRNA transfected PCs. (**B**) Nude mice were subcutaneously co-injected with HCC827 and scramble/IL32 targeting shRNA transfected PCs. 10 days post-injection, tumor-bearing mice were then treated with placebo or Osimertinib. (**C**) Line graph shows the tumor growth over time in each group. (**D, E**) Bar chart shows the final tumor weight or volume in each group. NS: non-significant difference. ***p < 0.001. Scale bar in (**B**) represents 1 cm. (**A**) Student’s t-test. (**C**) Two-way ANOVA. (**D, E**) One-way ANOVA.

**Table S1. Characteristics of a cohort of 20 NSCLC patients with EGFR mutations, all treated with third-generation TKI drugs.**

| Characteristics | No. of patients (%) |
| --- | --- |
| Gender  Male  Female  Age/years  Median  Range  EGFR^MUT^  T790M  L858R  delE746_A750  TNM stage  I-II  III-IV  Progression free survival  Median  <15 months  ≥15 months | 11 (55%)  9 (45%)  60  43-75  11 (55%)  6 (30%)  3 (15%)  0 (0%)  100 (100%)  16.5  10 (50%)  10 (50%) |

**Table S2. Sequences of siRNA/shRNA used in this study.**

| **Primer Name** | **（5'-3'）** |
| --- | --- |
| hs-STAT3-si-1-F | CCCUGAGCUAGUGACUAAAdTdT |
| hs-STAT3-si-1-R | UUUAGUCACUAGCUCAGGGdTdT |
| hs-STAT3-si-2-F | CGUCAUUAGCAGAAUCUCAdTdT |
| hs-STAT3-si-2-R | UGAGAUUCUGCUAAUGACGdTdT |
| hs-ETS1-si-1-F | CUUAUGAAGAUCCUCGAAUdTdT |
| hs-ETS1-si-1-R | AUUCGAGGAUCUUCAUAAGdTdT |
| hs-ETS1-si-2-F | CGCUAUACCUCGGAUUACUdTdT |
| hs-ETS1-si-2-R | AGUAAUCCGAGGUAUAGCGdTdT |
| hs-IL32-si-1-F | GGGAGAGCUUUUGUGACAAdTdT |
| hs-IL32-si-1-R | UUGUCACAAAAGCUCUCCCdTdT |
| hs-IL32-si-2-F | GUGACAAGGUCAUGAGAUGGUdTdT |
| hs-IL32-si-2-R | ACCAUCUCAUGACCUUGUCACdTdT |
| hs-YY1-si-1-F | CGACGACUACAUUGAACAAdTdT |
| hs-YY1-si-1-R | UUGUUCAAUGUAGUCGUCGdTdT |
| hs-YY1-si-2-F | GAUGAUGCUCCAAGAACAAdTdT |
| hs-YY1-si-2-R | UUGUUCUUGGAGCAUCAUCdTdT |
| shIL32-2 | GTGACAAGGTCATGAGATGGT |

**Table S3. The RT-PCR primer sequences utilized in this study.**

| **Gene** | **Forward primers (5'-3')** | **Reverse primers (5'-3')** |
| --- | --- | --- |
| IL-32 | TGGCGGCTTATTATGAGGAGC | CTCGGCACCGTAATCCATCTC |
| CXCL12 | ATTCTCAACACTCCAAACTGTGC | ACTTTAGCTTCGGGTCAATGC |
| IL-22 | CCTTCCCCAGTCACCAGTTG | TGCGGTTGGTGATATAGGGC |
| IL-17A | AGATTACTACAACCGATCCACCT | GGGGACAGAGTTCATGTGGTA |
| CXCL5 | AGCTGCGTTGCGTTTGTTTAC | TGGCGAACACTTGCAGATTAC |
| YY1 | AAGAGCGGCAAGAAGAGTTAC | CAACCACTGTCTCATGGTCAATA |
| STAT3 | CAGCAGCTTGACACACGGTA | AAACACCAAAGTGGCATGTGA |
| EGFR | AGGCACGAGTAACAAGCTCAC | ATGAGGACATAACCAGCCACC |
| ETS1 | GATAGTTGTGATCGCCTCACC | GTCCTCTGAGTCGAAGCTGTC |
| YY1-proximal site | TCGAGACTCCTTCATCAACA | TGGATAACCACTCATCTACTTT |
| YY1-distal site | ACCCTATTTCAATATGACTGGTGTC | CTGGGATTCTGTTCAGGTTTCT |
